# Structure and activation of the TSH receptor transmembrane domain

**DOI:** 10.1007/s13317-016-0090-1

**Published:** 2016-12-05

**Authors:** Ricardo Núñez Miguel, Jane Sanders, Jadwiga Furmaniak, Bernard Rees Smith

**Affiliations:** FIRS Laboratories, RSR Ltd, Parc Ty Glas, Llanishen, Cardiff, CF14 5DU UK

**Keywords:** Graves’ disease, Glycoprotein hormones, Glycoprotein hormone receptors, TSHR structure, Transmembrane domain structure, TSHR activation

## Abstract

**Purpose:**

The thyroid-stimulating hormone receptor (TSHR) is the target autoantigen for TSHR-stimulating autoantibodies in Graves’ disease. The TSHR is composed of: a leucine-rich repeat domain (LRD), a hinge region or cleavage domain (CD) and a transmembrane domain (TMD). The binding arrangements between the TSHR LRD and the thyroid-stimulating autoantibody M22 or TSH have become available from the crystal structure of the TSHR LRD–M22 complex and a comparative model of the TSHR LRD in complex with TSH, respectively. However, the mechanism by which the TMD of the TSHR and the other glycoprotein hormone receptors (GPHRs) becomes activated is unknown.

**Methods:**

We have generated comparative models of the structures of the inactive (TMD_In) and active (TMD_Ac) conformations of the TSHR, follicle-stimulating hormone receptor (FSHR) and luteinizing hormone receptor (LHR) TMDs. The structures of TMD_Ac and TMD_In were obtained using class A GPCR crystal structures for which fully active and inactive conformations were available.

**Results:**

Most conserved motifs observed in GPCR TMDs are also observed in the amino acid sequences of GPHR TMDs. Furthermore, most GPCR TMD conserved helix distortions are observed in our models of the structures of GPHR TMDs. Analysis of these structures has allowed us to propose a mechanism for activation of GPHR TMDs.

**Conclusions:**

Insight into the mechanism of activation of the TSHR by both TSH and TSHR autoantibodies is likely to be useful in the development of new treatments for Graves’ disease.

**Electronic supplementary material:**

The online version of this article (doi:10.1007/s13317-016-0090-1) contains supplementary material, which is available to authorized users.

## Introduction

The thyroid-stimulating hormone (TSH) receptor (TSHR) is a class A G protein-coupled receptor (GPCR) and is the target autoantigen in Graves’ disease [1, 2]. Patients with Graves’ disease develop autoantibodies that bind the extracellular domain (ECD) of the TSHR and activate the receptor. The autoantibodies mimic the action of TSH causing stimulation of thyroid hormone synthesis by thyroid cells, leading to hyperthyroidism in Graves’ disease [1, 2].

GPCRs constitute a large superfamily of integral membrane protein receptors. The first three-dimensional structure of a complete GPCR (bovine rhodopsin) [3] was solved in 2000. Since then a number of GPCR structures have been solved by experimental methods, published and deposited in the Protein Data Bank (PDB). All GPCR structures share a core of seven membrane-spanning helices. The major differences between different GPCRs are observed in the relative positions and contacts of the helices with respect to each other and the length and structures of their N termini, intracellular loops and extracellular loops. As the number of available experimental GPCR structures increases, the homology or comparative modelling methods can be used to obtain reliable models of the structures of other GPCRs with unsolved structures [4].

The TSHR belongs to the glycoprotein hormone receptor (GPHR) subfamily, of the leucine-rich repeat-containing GPCR (LRG) family, of class A (or rhodopsin like) GPCR [5]. The structure of the TSHR, as well as the other GPHRs, is composed of a large amino-terminal extracellular domain (ECD) and a transmembrane domain (TMD). The TSHR ECD contains an N-terminal domain, a leucine-rich repeat domain (LRD) and a hinge region or cleavage domain. The TMD contains the typical seven transmembrane helices of GPCRs, an eighth helix parallel to the membrane and a C-terminal tail. The crystal structures of the LRD of the human (h) TSHR in complex with the TSHR-stimulating human monoclonal autoantibody (hMAb) M22 [6] and with the TSHR-blocking hMAb K1–70 [7] are available. Also, the crystal structure of the LRD and the ECD of the human FSH receptor (FSHR) bound to hFSH has been determined [8, 9]. No experimental structures of the TMD of the TSHR are available, although several models of the structure of the TSHR TMD have been published [10–18].

The availability of three GPCR crystal structures in their fully active conformation, β2-adrenergic receptor, rhodopsin (metarhodopsin II) and M2 muscarinic acetylcholine receptor, has provided some insight into GPCR activation [19–21]. Here, we present the comparative models of the structures of the TMD of the TSHR in its active and inactive conformations based on the same three GPCR structures for which both, active and inactive crystal structures are available, i.e. β2-adrenergic receptor, rhodopsin and M2 muscarinic acetylcholine receptor [19–24]. We also modelled structures of the TMDs of the FSHR and LH receptor (LHR) in their active and inactive conformations. In addition, we produced a model of the structure of the TSHR TMD in its inactive conformation based on 16 GPCR inactive structures. These structures have allowed us to propose an activation mechanism for the TSHR TMD.

## Methods

Three theoretical models of the structure of the TSHR TMD have been obtained by comparative modelling using the program MODELLER [25] within the Discovery Studio 3.5 suite of software (DS3.5) (http://accelrys.com/products/discovery-studio/). The TSHR TMD structure (TMD_I) was predicted using 16 homologous GPCR experimental structures in their inactive state (Table 1). The TSHR TMD structure (TMD_In) was predicted using three homologous GPCR crystal structures (β2-adrenergic receptor, metarhodopsin II and M2 muscarinic acetylcholine receptor) in their inactive state (Table 1). The TSHR TMD structure (TMD_Ac) was predicted using the same three homologous GPCR experimental structures as for the structure of the TMD_In, but, in this case, in their active state (Table 1). In addition, structures of the TMDs of the FSHR and LHR in both active and inactive conformations were obtained using the same GPCR templates used for the TSHR TMD structures. In the case of all homologous GPCRs used, the coordinates of T4-lysozyme, antibodies/nanobodies and G proteins/peptides were removed. In addition, when the sequences of the segments of the GPCRs and the TSHR TMD had different lengths, the sequences of the homologous GPCRs were adjusted. When the sequence of the TSHR TMD showed an insertion compared to the sequences of the templates, one or two residues of the templates were deleted at each side of the insertion. When the sequence of the TSHR TMD showed a deletion compared to the sequences of the templates, the additional residues were deleted from the templates plus one or two residues of the templates at each side of the deletion. The deletion of one or two residues at the boundaries of the insertions/deletions was dependent on the structures of the templates. The crystal structure of the extracellular loop 1 (ECL1) of the human smoothened receptor (Table 1) was used to predict the structure of the ECL1 of the three modelled structures of the TSHR TMD (Table 1). The amino acid sequence identities between the homologous GPCR and the TSHR TMD are shown in Table 1. The same homologous GPCR structures used for modelling the structure of the TSHR TMD were used for modelling the active and inactive conformations of the FSHR and LHR TMDs.

**Table 1:** Homologous GPCR experimental structures used for the comparative modelling of the TSHR, FSHR and LHR

Receptor	Species	PDB-Id	State	Resolution	TMD identity^a^ (%)	Used for the modelling of			References
						TMD_I	TMD_In	TMD_Ac	
CXC chemokine receptor type 1	Human	2LNL	Inactive	NMR	20.7	✓	✖	✖	[50]
β1-Adrenergic receptor	Turkey	2VT4	Inactive	2.7 Å	18.4	✓	✖	✖	[51]
Rhodopsin	Squid	2Z73	Inactive	2.50 Å	17.6	✓	✖	✖	[52]
Adenosine A_2A_ receptor	Human	3EML	Inactive	2.60 Å	21.0	✓	✖	✖	[53]
CXC chemokine receptor type 4	Human	3ODU	Inactive	2.50 Å	19.5	✓	✖	✖	[54]
Histamine H1 receptor	Human	3RZE	Inactive	3.10 Å	18.0	✓	✖	✖	[55]
Sphingosine 1-phosphate receptor 1	Human	3V2Y	Inactive	2.80 Å	17.5	✓	✖	✖	[56]
Proteinase-activated receptor 1	Human	3VW7	Inactive	2.20 Å	17.6	✓	✖	✖	[57]
M3 muscarinic acetylcholine receptor	Rat	4DAJ	Inactive	3.40 Å	20.5	✓	✖	✖	[58]
κ-Opioid receptor	Human	4DJH	Inactive	2.90 Å	18.0	✓	✖	✖	[59]
μ-Opioid receptor	Mouse	4DKL	Inactive	2.80 Å	18.8	✓	✖	✖	[60]
Nociceptin/orphanin FQ receptor	Human	4EA3	Inactive	3.01 Å	20.7	✓	✖	✖	[61]
δ-Opioid receptor	Mouse	4EJ4	Inactive	3.40 Å	20.0	✓	✖	✖	[62]
Rhodopsin	Bovine	1U19	Inactive	2.20 Å	17.7	✓	✓	✖	[22]
β2-Adrenergic receptor	Human	2RH1	Inactive	2.40 Å	21.4	✓	✓	✖	[23]
M2 muscarinic acetylcholine receptor	Human	3UON	Inactive	3.00 Å	19.7	✓	✓	✖	[24]
Metarhodopsin II	Bovine	3PQR	Active	2.85 Å	17.7	✖	✖	✓	[19]
β2-Adrenergic receptor-Gs protein complex	Human	3SN6	Active	3.20 Å	21.4	✖	✖	✓	[20]
M2 muscarinic acetylcholine receptor	Human	4MQS	Active	3.50 Å	17.7	✖	✖	**✓**	[21]
Smoothened receptor	Human	4LKV	Inactive	2.45 Å	–	ECL-1			[63]

^a^Amino acid sequence identity between the TSHR TMD and the homologous GPCRs

Three initial sequence alignments of the amino acid sequences of the GPCR used as templates were obtained using the program ClustalW [26]. The initial sequence alignments were manually modified based on structural alignments and superimpositions of coordinates of the homologous GPCRs using DS3.5. Three initial alignments of the amino acid sequence of the TSHR TMD (or the FSHR and LHR TMDs) with the previous alignments of GPCRs homologues were obtained using the program ClustalW. The alignments were manually modified to correct defects from the automatic alignment method of ClustalW.

The structures of the TSHR TMD (or the FSHR and LHR TMDs) predicted by MODELLER were validated with the ‘Check Structure’ and ‘Profiles-3D’ functionalities of DS3.5. The alignments were manually modified to improve validation results, and the modelling and validation processes repeated until models with good geometry and conformations were obtained. Finally, some short sequence segments were remodelled by the “Loop Refinement” functionality within DS3.5 to correct structural defects.

## Results

At the time of the study, the experimental structures of 18 different inactive GPCRs and three structures in the active state were available from the PDB. The structures of 16 GPCRs in an inactive state were used as templates to build an inactive structure of the TSHR TMD (TMD_I) (Table 1). An active structure of the TSHR TMD (TMD_Ac) was built based on the only three templates of fully active GPCRs. Furthermore, an inactive TSHR (TMD_In) structure was built based on the same three templates used for TMD_Ac, but in their inactive states (Table 1).

The TSHR TMD ECL1 is 14 residues long, whereas the lengths of the ECL1 of the templates used for modelling range from five to eight residues. Therefore, the ECL1 from the smoothened receptor, which is 26 residues long, was used for modelling the structure of the ECL1 of the TSHR TMD. The structure of the ECL1 of the smoothened receptor shows a short α helix at its N terminus followed by a β turn type II, then a segment bound to the top of the TMD structure and finished by a β turn type I. The segment bound to the top of the TMD structure has been used in the modelling of the structure of the TSHR TMD ECL1 (Supplementary Fig. 1).

The ECL2 of the TSHR TMD is 23 residues long and therefore challenging for predicting its structure in silico. A sequence alignment of the ECL2 of the 16 GPCR templates, used for comparative modelling, with that of the TSHR TMD (Supplementary Fig. 1) identified bovine rhodopsin as having the highest amino acid sequence identity (32%). The structure of the ECL2 of bovine rhodopsin shows a small two-stranded β sheet placed on the top of the TMD structure, forming a “lid”, followed by a segment going up and finishing in a small α helix. The structure of the TSHR TMD ECL2 may also act as a “lid”, because, as in the case of rhodopsin, no ligand is needed to access the space between the transmembrane helices. Accordingly, rhodopsin was selected as the most appropriate template for modelling the structure of the ECL2 of the TSHR TMD.

The ICL3 shows great variation in length among the GPCRs, from as short as five residues to a complete domain present within the loop sequence in wild-type proteins (Supplementary Fig. 1). The sequence of the TSHR TMD ICL3 is one of the shortest, having five or seven residues depending on possible variations in determining the beginning and the end of the loop. For the purpose of modelling, the ICL3 segments of GPCR templates have been adjusted to match the length of TSHR TMD ICL3, except for squid rhodopsin template. The transmembrane helices (TMs) 5 and 6 flanking the ICL3 of squid rhodopsin are longer than those of the other GPCRs. The intracellular parts of TM5 and 6 and ICL3 of squid rhodopsin were kept to obtain a better definition of the structures of the TSHR TM5 and 6.

### Comparative modelling of the structure of the inactive TSHR TMD_I

The 16 structures of the inactive GPCRs with the highest amino acid sequence identity (cutoff at 17.5%) compared with the TSHR TMD sequence were used to model the structure of TSHR TMD_I (Table 1). The amino acid sequence alignment between the 16 homologous GPCRs used for modelling and the TSHR TMD is shown in Supplementary Fig. 1, together with the location and number of residues that have been removed from the structures of the homologous GPCRs, for the purpose of the study, and the residues that are not visible in the experimental structures.

The amino acid sequences of GPCRs show some highly conserved motifs. One functionally important motif corresponds to the sequence E/D^3.49^RY^3.51^ in TM3 (superscripts refer to Ballesteros–Weinstein numbering [27], Supplementary Fig. 2). The Arg residue of this motif forms a salt bridge (ionic lock) with Glu^6.30^ of TM6 that stabilizes the GPCR inactive state [28]. The TSHR shows the sequence ERW (Glu518^3.49^, Arg519^3.50^, Trp520^3.51^) at the equivalent position and an Asp^6.30^ at position 619 of TM6 (Supplementary Figs. 1 and 2), and in our model they form the corresponding salt bridge of the “ionic lock”. Furthermore, a conserved direct or water-mediated hydrogen bond network linking Asp^2.50^ of the NLxxxD motif in TM2 with Trp^6.48^ of the CWxP motif in TM6 is important for maintaining the inactive conformation [29]. The sequence of the TSHR shows the conserved motif 455-NLxxxD-460 in TM2 but not in TM6, where the corresponding sequence is 636-CMxP-639 (Supplementary Fig. 1). A hydrogen bond network is not observed in the structure of the TMD_I; however, a water-mediated hydrogen bond network cannot be ruled out as water molecules were not included in the model.

**Fig. 1:**
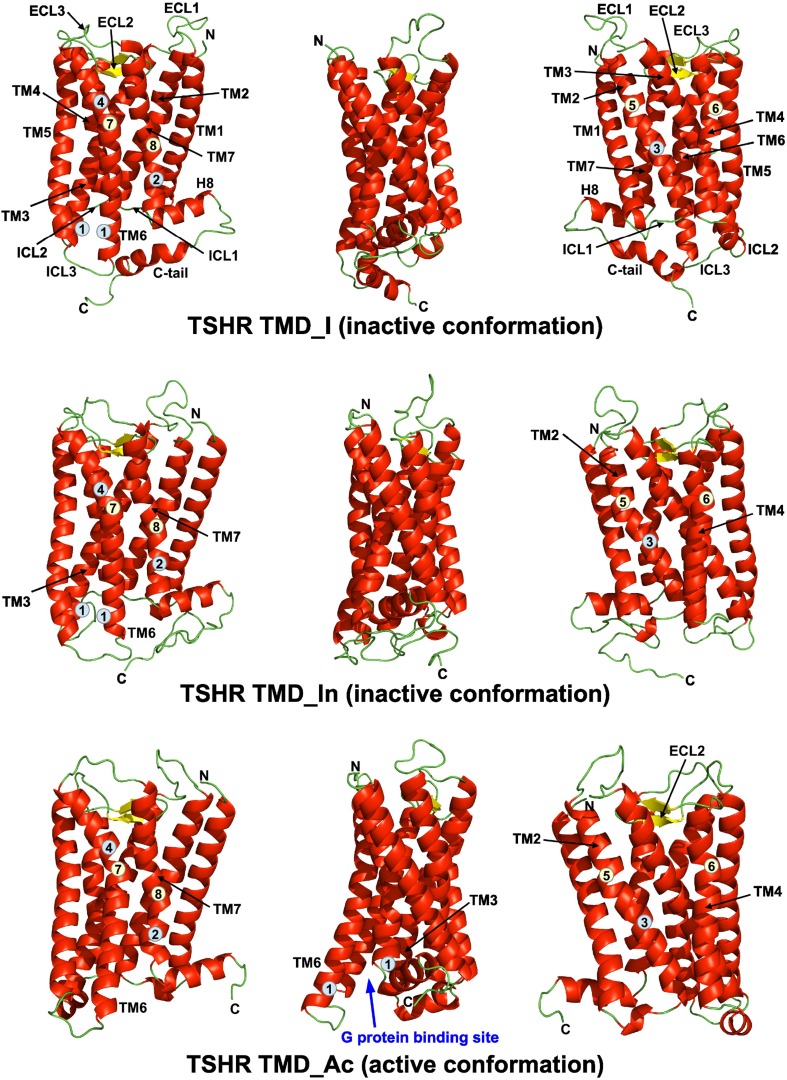
Comparative models of the structure of the transmembrane domain (TMD) of the thyroid-stimulating hormone receptor (TSHR) in three different orientations related by 90° rotations along a *vertical axis*. TMD_I is based on 16 G protein-coupled receptor (GPCR) structures in their inactive conformations. TMD_In is based on three GPCR structures in their inactive conformations. TMD_Ac is based on the same three GPCRs as TMD_In, but in their active conformations. Transmembrane helices (TM), extracellular loops (ECL), intracellular loops (ICL), C-terminal tail (C-tail) and N- and C-termini (N, C) are marked. Sequence motifs (*blue background circles*) and structural features (*yellow background circles*) of the transmembrane domain of the TSH receptor in their active and inactive conformations are shown. Ionic lock (*1*), N^674^PxxY^678^ motif (*2*) in TM7, N^455^LxxxD^460^ motif (*3*) in TM2 and C^636^MxP^639^ motif (*4*) in TM6. Alpha bulge (*5*) in TM2, proline distortion (*6*) in TM4, proline kink (*7*) in TM6 and proline kink (*8*) in TM7. The G-protein binding site is clearly visible at the cytoplasmic end of the active conformation. Reproduced with permission from copyright holder RSR Ltd

Some GPCR structures have an α-bulge in the middle of TM2. It was proposed that α-bulges are generated by adjacent prolines; however, there are exceptions [30]. For example, the muscarinic acetylcholine receptor M3 (mAChR M3, PDB-Id 4DAJ, Table 1) has the α-bulge in its TM2 in the absence of an adjacent proline. The sequence alignment between the TSHR TMD and its homologous GPCRs (Supplementary Fig. 1) suggests the presence of an α-bulge in the structure of TM2 of the TSHR TMD, and even though no proline is present the α-bulge was included in our model. A proline distortion is observed close to the extracellular end of TM4 of most GPCR structures. The presence of a proline at the same position in the amino acid sequence of the TSHR TMD TM4, Pro556^4.60^ (Supplementary Fig. 1), is a good indication that a similar distortion is likely to occur in the structure of TM4 of the TSHR TMD and consequently the model shows the distortion. Furthermore, out of the 18 available TMD structures, only the sphingosine 1-phosphate receptor 1 (S1P1, PDB-Id 3V2Y) does not present an α-bulge in its TM5 or a proline, Pro^5.50^, in its TM5. The sequence alignment between the TSHR TMD and its homologous GPCRs (Supplementary Fig. 1) shows that no proline is present in TM5 as in the case of the S1P1, and therefore TM5 of the TSHR TMD does not present an α-bulge (Fig. 1). A proline kink is observed in the structure of the TSHR TMD TM6. A similar proline kink is observed in the structures of all GPCR used as templates for modelling (Fig. 1 and Supplementary Fig. 1). A similar situation is observed in TM7, where all GPCR templates and the TSHR TMD show a proline kink (Fig. 1 and Supplementary Fig. 1).

For the modelling of the structure of the TSHR TMD_I ECL2, we have used the ECL2 structure of rhodopsin and ECL2 structures similar to that of rhodopsin found in five GPCRs. (Supplementary Fig. 1). The conserved disulphide bond between the cysteines of the ECL2 and TM3 is present in the structure of the TSHR TMD_I. The ICL2 of the TSHR TMD_I shows a small α helix similar to that observed in some of the structures of GPCRs used as templates for modelling (Fig. 1 and Supplementary Fig. 1). For ICL3, the whole structure of squid rhodopsin ICL3 was used for modelling. The structures of the cytoplasmic ends of TM5 and TM6 of squid rhodopsin are about two-and-a-half helix turns longer for each helix compared to the other GPCRs used as templates. Using squid ICL3 for modelling helped in defining good helical structures of the cytoplasmic ends the TSHR TM5 and TM6 helices.

### Comparative modelling of the structure of the inactive TSHR TMD_In (three homologues)

A comparative model of the structure of the inactive TSHR TMD (TMD_In) has been obtained based on the structures in an inactive conformation of the same three GPCRs for which their active conformation structures are available (β_2_-adrenoceptor, metarhodopsin II and M2 muscarinic acetylcholine receptor) (Table 1). The amino acid sequence alignment between the three homologous GPCRs used for modelling and the TSHR TMD is shown in Supplementary Fig. 3, together with the location and number of residues that have been removed from the structures of the homologous GPCRs and the residues that are not visible in the experimental structures.

In the model of the structure of TSHR TMD_In (Fig. 1), the Arg residue of the conserved motif 518-E/D^3.49^RY^3.51^ (Glu/Asp518, Arg519, Tyr520) is involved in an electrostatic interaction with Asp619^6.30^ of TM6, forming an “ionic lock” that stabilizes the inactive conformation (Fig. 1). In contrast, Tyr678^7.53^ from the conserved motif N^7.49^PxxY^7.53^ in TM7 is ~11 Å away from Tyr601^5.58^ in TM5 and this distance makes formation of a water-mediated hydrogen bond impossible, consistent with an inactive conformation. Furthermore and similarly to the structure of the TMD_I, the hydrogen bond network connecting Asp460^2.50^ of the 455-NLxxxD-460 motif in TM2 with Met637^6.48^ of the 636-CMxP-639 motif in TM6 is not observed in the modelled structure of the TMD_In. As the model does not include water molecules, a water-mediated hydrogen bond network cannot, however, be ruled out.

Similar to the structure of the TSHR TMD_I, the structure of the TMD_In shows an α-bulge in the middle of TM2 but not in TM5 (Fig. 1). In addition, similar proline distortion to that observed close to the extracellular end of TM4 of the TMD_I is observed in the structure of the TMD_In (Fig. 1). Proline kinks are also observed in the structures of TM6 and TM7 of the TSHR_In (Fig. 1) as in the case of the TMD_I. In addition, the conserved disulphide bond between cysteines of the ECL2 and TM3 is also observed. The ICL1 of the TSHR TMD_In shows a small α helix similar to that observed in two of the three structures of GPCRs used as templates for modelling (Fig. 1 and Supplementary Fig. 1). In contrast to the TMD_I structure, no small α helix is observed in the structure of the ICL2 of the TMD_In.

### Comparative modelling of the structure of the active TSHR TMD_Ac (three homologues)

The structure of the TSHR TMD in its active conformation (TMD_Ac) has been obtained by comparative modelling based on the structures of the only three GPCRs for which fully active conformation structures are available (β_2_-adrenoceptor, metarhodopsin II, and M2 muscarinic acetylcholine receptor) (Table 1). The same three GPCRs in the inactive conformations were used for modelling the structure of the inactive TSHR TMD (TMD_In). This allowed a comparison of the active and inactive conformations of the TSHR TMD. The amino acid sequence alignment between the three homologous GPCRs used for modelling and the TSHR TMD is shown in Supplementary Fig. 4, together with the location and number of residues that have been removed from the structures of the homologous GPCRs and the residues that are not visible in the experimental structures.

In the model of the structure of the TMD_Ac (Fig. 1), the Arg519^3.50^ residue of the conserved motif E/DRY is ~13 Å away from Asp619^6.30^ of TM6 breaking the “ionic lock” as expected for an active conformation (Fig. 1). Another functionally important GPCR motif, N^7.49^PxxY^7.53^ in TM7, makes a water-mediated hydrogen bond with the highly conserved Tyr^5.58^ in TM5 in the structures of activated β_2_-adrenoceptor and metarhodopsin II. The water-mediated hydrogen bond, only possible in the active state, may contribute to active state stability in GPCRs, serving as an active state counterpart to the ‘ionic lock’ that stabilizes the inactive state [31]. The TSHR shows the exact amino acid sequence, 674-NPxxY-678, of this highly conserved motif and also shows a tyrosine in TM5 at position 5.58 (Tyr601) (Supplementary Figs. 1 and 2). TSHR TMD_Ac Tyr678^7.53^ from the NPxxY motif is at a non-interacting distance of ~4.6Å from Tyr601^5.58^ in TM5. This distance is similar to that observed in the structure of metarhodopsin II (~5.0 Å) which has the two tyrosines hydrogen bonded through a water molecule stabilizing the active conformation. Although the model of the structure of TMD_Ac does not show positions of water molecules, it is expected that the TMD_Ac equivalent tyrosines would be involved in a water-mediated hydrogen bond.

In a similar way to the structures of TSHR TMD_I and TMD_In, the structure of TMD_Ac shows an α-bulge in the middle of TM2 (Fig. 1), but not in the structure of TM5. In addition, similar proline distortion to that seen close to the extracellular end of TM4 of the TMD_I and TMD_In is observed in the structure of the TMD_Ac (Fig. 1). Finally, proline kinks are also observed in the structures of TM6 and TM7 of the TSHR_Ac (Fig. 1) as in the case of the TMD_I and TMD_In. In addition, the conserved disulphide bond between cysteines of the ECL2 and TM3 is also observed. The ICL1 and ICL2 of the TSHR TMD_Ac show small α helices similar to those observed in some of the structures of GPCRs used as templates for modelling (Fig. 1 and Supplementary Fig. 1).

### Comparative modelling of the structures of the TMDs of the FSHR and LHR

The structures of the active and inactive conformation of the FSHR TMD and LHR TMD were obtained by comparative modelling based on the same GPCR structures, in their inactive and fully active conformations, used for modelling the TSHR TMD_In and TMD_Ac. The structures of the inactive and active conformations of the TMDs of the FSHR and LHR are highly similar to the structures of the inactive and active conformations of the TSHR TMD, respectively.

All major GPCR-conserved motifs show the same sequences in the three GPHRs, i.e. NLxxD^2.50^, ERW^3.51^, CMxP^6.50^ and NPxxY^7.53^. In addition, similar to the structures of the TSHR TMD, an α bulge is observed in the structures of TM2, but not TM5 of the FSHR and LHR TMDs. Similar proline distortion in TM4 and proline kinks in TM6 and TM7 are observed in the structures of the three GPHRs. Functionally important interactions are similar in the three GPHRs. For example, the ionic lock observed in most GPCRs, a salt bridge between Arg^3.50^ in TM3 and Asp/Glu^6.30^ in TM6, is observed in the inactive conformations of the three GPHRs (Arg467 and Asp567 in the FSHR, Arg464 and Asp564 in the LHR, and Arg519 and Asp619 in the TSHR), but it is broken in the three active conformations. Similarly, the possible water-mediated hydrogen bond between Tyr^5.58^ in TM5 and Tyr^7.53^ in TM7, observed in the active conformation of the TSHR TMD, is also possible in the active conformations of the FSHR TMD and LHR TMD, but not possible in the three inactive conformations.

## Discussion

### Comparative modelling

It has been proposed that comparative modelling using multiple templates would produce more accurate models compared to the models based on a single template [32, 33] provided that the templates have similar levels of sequence identity with the target sequence. In particular, a model based on a single template would be highly similar to the chosen template and may not be the best representation of the target structure. In contrast, multi-template modelling would produce a model with an average structure of the templates and after minimization would be expected to be a better representation of the target structure. In this study, we have produced a model of the structure of the inactive TSHR (TSHR TMD_I) based on 16 of the 18 GPCRs templates in their inactive states available at the time of the study. Consequently, the TSHR TMD_I model is likely to represent an accurate structure of the inactive TSHR TMD. However to compare the models of the inactive and active conformations of the TSHR (as well as FSHR and LHR), the models should be produced based on templates of the inactive and active conformations of the same GPCRs. Therefore, we have built comparative models of the inactive and active conformations of the three receptors based on the three GPCRs for which structures of both inactive and fully active conformations were available at the time of the study. The structures of partially active conformations were not considered as suitable templates as they present features of active conformations in the extracellular parts of the receptors and features of inactive conformations in the cytoplasmic parts. To date, a comparison of the inactive and active conformations of the GPHRs to infer an activation mechanism of the TMD has not been carried out. The majority of previously published models of the active state of GPHRs were based only on one template [11–13, 15–18]. In one study, a model of the TSHR TMD was produced based on 15 GPCRs in their inactive conformations, 3 GPCRs in partially active conformations and 1 in fully active conformation [14]. However, a receptor model obtained in this way would not show either inactive or active conformation. In addition, Schaarschmidt et al. [10] have obtained an active conformation of the TSHR TMD based on five partially active and two fully active GPCR template structures. A model of the active TSHR TMD, obtained in this way, is likely to represent the inactive conformation in its cytoplasmic region rather than the active conformation. In contrast, in our study we analysed the activation of the GPHR TMDs based on the TMD_In and TMD_Ac structures of the three receptors.

### TSHR TMD structure

The structures of the TSHR TMD (and the FSHR and LHR TMDs) are similar to those of typical GPCRs, with seven transmembrane helices, an eighth helix parallel to the membrane, three extracellular loops, three cytoplasmic loops and a C-terminal tail. The interactions between GPCR residues of conserved motifs are also observed in the structures of the TSHR TMD (and the FSHR and LHR TMDs) including a functionally important ‘ionic lock’ in the inactive state that is not present in the active state.

The structures of our three models of the TSHR TMD and the models of the FSHR and LHR TMDs show an α-bulge in the middle of TM2 (Fig. 1), but not in the structure of TM5, as previously predicted by Chantreau et al. [11]. GPHRs have an alanine at position 5.50 (Ballesteros–Weinstein numbering [27]) of TM5 in contrast to other GPCRs that have a conserved Pro in this position and consequently a kink in the structure of TM5. Therefore, the models of the structures of TM5 of GPHRs show a regular α helix without a kink, as predicted previously by Kleinau et al. [15] and Chantreau et al. [11]. TSHR mutation A593^5.50^P is likely to induce a kink in the TM5 structure that would probably affect folding of the mutated receptor. Indeed, the cell surface expression of TSHR A593P has been determined to be ~6% of TSHR wild type [15].

Strong hydrophobic interactions are observed in the GPHR subfamily between residues at positions 3.30 (Phe447 in FSHR, Phe444 in LHR and F499 in TSHR) and 4.58 (Leu502 in FSHR, Met499 in LHR and Leu554 in TSHR). These interactions are observed for both active and inactive conformations of GPHRs. However, equivalent interactions are not observed in the three GPCR crystallized in their active conformation, whereas four of the 18 GPCR inactive experimental structures used in this study (16 structures used as templates plus 2 structures not used as templates due to low amino acid sequence identity) show weak contacts between residues at positions 3.30 and 4.58. In contrast, the remaining 14 GPCR structures do not show this interaction. Consequently, as experimental structures of other members of the leucine-rich repeat-containing GPCR family are not available as yet, the hydrophobic interactions between residues at positions 3.30 and 4.58 observed in our models may be considered specific for the GPHR subfamily.

### TSHR TMD activation

The crystal structure of the FSHR ECD without the TMD, solved by Jiang et al. [9], shows the ECD in its active conformation. This indicates that the ECD can adopt the active conformation in the absence of the TMD. In addition, in most class A GPCRs the ECDs are not involved in activation, suggesting that the TMD is likely to activate independently of the ECD. According to this, the activation mechanism of GPHRs can be defined by the following independent steps: binding of the hormone or an agonist stimulating antibody to the LRD of the receptor’s ECD; activation of the ECD of the receptor; signal transduction from the ECD to the TMD; activation of the TMD; and activation of the G protein. In this study, we have analysed the step involving activation of the TMD of the receptors.

The available GPCR crystal structures mainly define three different conformations: an “inactive state” in which the receptor is bound to an antagonist or inverse agonist; an “agonist-bound state” but without the G protein, or a G-protein equivalent; and a “fully active state” in which the receptor binds to an agonist and to a G protein or a G-protein equivalent, forming a ternary complex. Intermediate conformations are observed between these three states. Agonist-unbound GPCRs usually show basal activity that is enhanced by agonist binding, reduced by inverse agonist binding, unaffected by neutral antagonists and increased by mutations that lead to constitutively active mutants (CAMs). CAMs displace the active–inactive equilibrium of GPCRs to the active state side [37]. Until 2002, constitutive activities had been observed in more than 60 wild-type GPCRs [34]. More recently, Martin et al. [35] have reported constitutive activity in 75% of the 40 orphan class A GPCR they studied. In the case of GPHRs, although the wild-type TSHR shows high levels of constitutive activity, the wild-type FSHR and LHR display little constitutive activity if any [36].

Mechanisms of activation of class A GPCRs have been unified by Tehan et al. [37]. Activation of GPCRs mostly results in a slight rotation and upward movement of TM3, a rotation of TM6 and inward movements of TM1, TM5 and TM7. The disruption of the ionic lock, between TM3 and TM6, may facilitate the movements of TM3 and TM6 during activation. However, more important is the hydrophobic rearrangement of residues Leu^3.43^, Phe^6.44^, Xxx^6.40^ (Xxx being one of the bulky hydrophobic amino acid, Ile, Leu, Val or Met) and the less important Xxx^6.41^, between TM3 and TM6 in the core of the receptor [37].

Figure 1 shows the structures of the three models of the TSHR TMD. The structure of the active conformation, TMD_Ac, shows a clear difference when compared with the inactive conformation, TMD_In. The G-protein binding site is evident in the structure of the TMD_Ac.

**Fig. 2:**
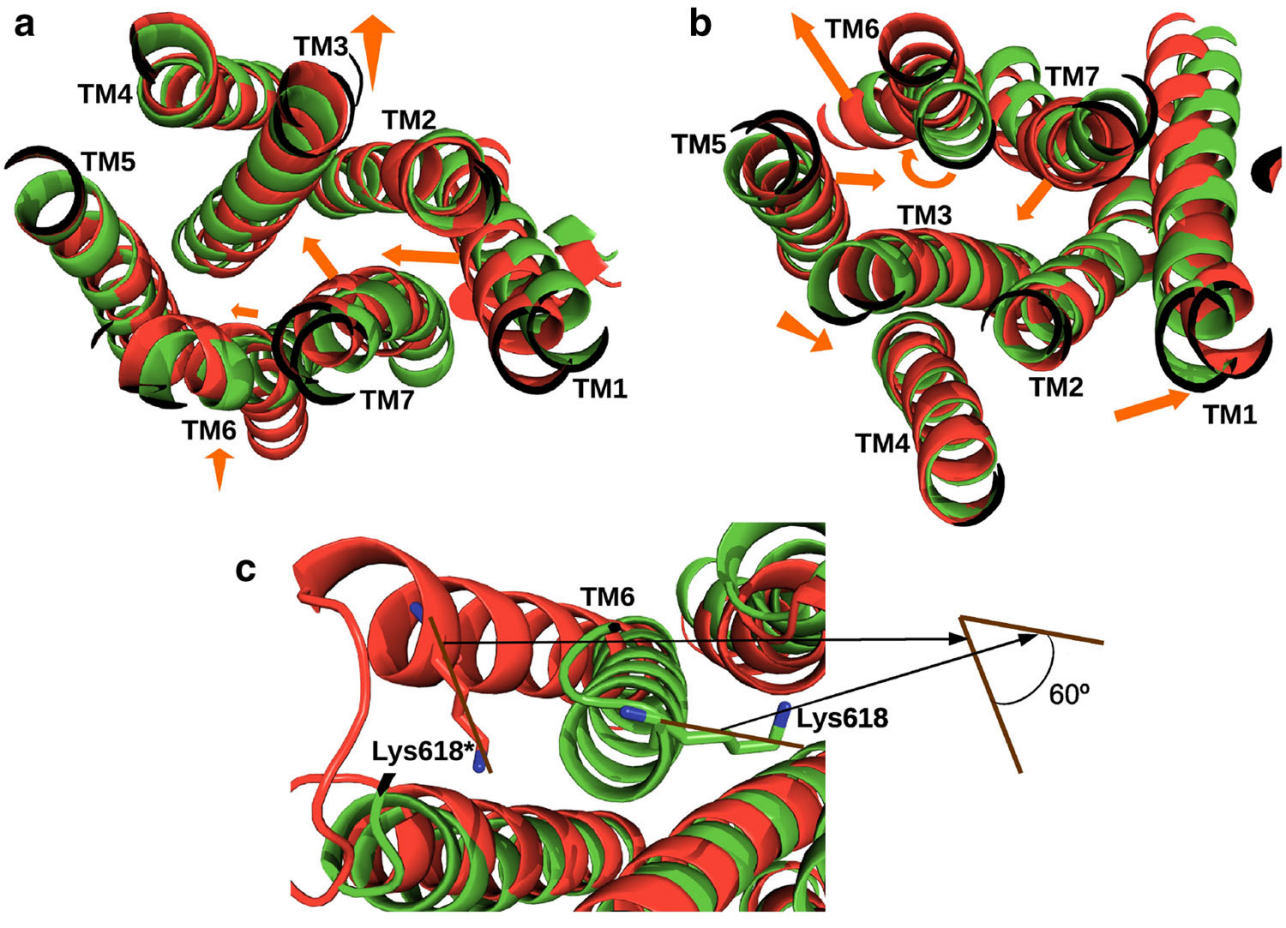
Movements of the transmembrane helices (TM) of the thyroid-stimulating hormone receptor (TSHR) transmembrane domain during the activation process. Orange arrows indicate direction of movement. **a** View from the extracellular side. **b** View from the intracellular side. **c** Rotation of TM6 shown by the position of Lys618 in the inactive and active states. Lys618 in the active state is marked with an *asterisk*. Reproduced with permission from copyright holder RSR Ltd

To show the conformational movements during the activation process of the TSHR TMD, a superimposition of the structures of TMD_In and TMD_Ac has been produced (Fig. 2). The movements during activation of the transmembrane helices at their extracellular ends involve (Fig. 2a) inward movements of TM1and TM7 towards the core of the helix bundle, upward movements of TM3 and TM6 and a small lateral movement of TM6. The movements of the intracellular ends of the transmembrane helices (Fig. 2b) involve outward movements of TM1 and TM6 away from the core of the helix bundle, inward movements of TM5 and TM7, a downward movement of TM3 and a rotation of TM6 (Fig. 2c). These movements are similar to those observed in other GPCRs [37].

Similar to other GPCRs, in the structure of the TSHR TMD, the ionic lock between Arg519^3.50^ in TM3 and Asp619^6.30^ in TM6 is present in the inactive state and absent in the active state. In the active state, Arg519^3.50^ is hydrogen bonded to Tyr601^5.58^ in TM5, whereas Asp619^6.30^ is hydrogen bonded to the backbone carbonyl oxygen of Val608^5.65^ in TM5. Furthermore, in the inactive TSHR TMD structure, Asn674^7.49^ in TM7, is hydrogen bonded to Asp460^2.50^ in TM2, and the latter is also hydrogen bonded to Ser671^7.46^ in TM7. In the TSHR TMD active state, Asn674^7.49^ remains hydrogen bonded to Asp460^2.50^ with the latter also hydrogen bonded to Ser671^7.46^, but both hydrogen bonds are stronger than in the inactive conformation. In addition, in the active state, Asn674^7.49^ and Asp460^2.50^ are both hydrogen bonded to Ser508^3.39^, in TM3. A proposed direct interaction between Asp633^6.44^ and Asn674^7.49^, based on mutations experiments [38], is not observed in the structure of the model of the inactive TSHR TMD; however, a water-mediated hydrogen bond is possible (distance of 4.6 Å). In the structure of the inactive TSHR TMD model, Asp633 is hydrogen bonded to the neighbouring Asn670^7.45^ not Asp674^7.49^ (Supplementary Table 3).

Activation of the TSHR TMD involves rearrangements of hydrophobic residues. In the inactive state, TSHR Leu512^3.43^ in TM3 makes hydrophobic contacts with both Leu629^6.40^ and Ile630^6.41^ in TM6, whereas in the active state it makes only one hydrophobic contact with Leu456^2.46^ in TM2. Instead of Pro^5.50^ in TM5 that is highly conserved in most GPCRs, the GPHRs have an alanine (Ala593^5.50^). TSHR TMD activation involves the breaking of the hydrophobic interaction between Ala593^5.50^ (in TM5) and Val509^3.40^ (in TM3) and the formation of a new hydrophobic interaction between Ala593^5.50^ and Thr513^3.44^ (in TM3). In addition, GPHRs have a charged Asp amino acid (Asp633^6.44^) in TM6 instead of the highly conserved hydrophobic Phe^6.44^ observed in most GPCRs. In the inactive state, TSHR Asp633^6.44^ makes a hydrogen bond with Asn670^7.40^ (in TM7), a possible water-mediated hydrogen bond with Asn674^7.44^ (in TM7) [39] and an induced dipole and hydrophobic interactions with Leu512^3.43^, from TM3. In the TSHR TMD active state, Asp633^6.44^ also makes an induced dipole interaction with Leu512^3.43^ (in TM3) and a polar interaction with the backbone carbonyl oxygen of Leu629^6.40^ (in TM6). Thus, activation of the TSHR TMD involves breaking of the interactions of Asp633^6.44^ in TM6 with Asn670^7.40^ and Asn674^7.44^ in TM7. Our models of active and inactive conformations of the three GPHR TMDs show that activation of the FSHR TMD and LHR TMD is likely to involve similar interaction rearrangement to those observed for the TSHR TMD.

### Activating mutations in the TMD of GPHRs

The GPHR family contains the functionally important Leu^3.43^ and Asp^6.44^ that stabilize the inactive state of the receptor, while GPCRs have Phe^6.44^ instead of Asp^6.44^ [37]. Three naturally occurring TSHR Leu^3.43^ polar mutants (Q/N/R) that increase receptor constitutive activity [40–42] show reduced inter-helical packaging. In addition, four natural TSHR Asp^6.44^ mutants (A/E/H/Y), also associated with increased receptor constitutive activity [43–45], break or modify the interaction between Asp^6.44^ and Asn^7.49^. Mutational studies have shown that the interaction between Asp^6.44^ and Asn^7.49^ stabilize the inactive state of the TSHR TMD [38]. In the case of the LHR, one naturally occurring Leu^3.43^ mutant [46] and three Asp^6.44^ mutants [47–49] that increase the constitutive activity of the receptor have been reported. In contrast, no naturally occurring activating mutants are reported at present for hFSHR at Asp^6.44^. Our models are consistent with the observations from naturally occurring activating mutations as described above (TSHR TMD activation).

Tables 2, 3 and 4 show a list of GPHR CAMs taken from the literature, whereas Supplementary Tables 1–3 list the main interactions involving the mutated residues of the CAMs as observed in the comparative models of the structures of the GPHRs. For example, the model of the structure of the FSHR shows that Met401^2.43^ makes a hydrophobic interaction with Ile463^3.46^ in the inactive conformation and an induced dipole interaction with Arg467^3.50^ in the active conformation (Supplementary Table 1). Substitution of Met401 for a Thr in the FSHR increases the constitutive activity of the mutant 3.6-fold compared to wild type (Table 2). The new Thr at position 2.43 of the mutated receptor may lose the hydrophobic interaction with Ile463 in the inactive conformation or change it to an induced dipole interaction. In addition, the induced dipole interaction of Met401 with Arg467 in the active conformation is likely to result in hydrogen bond formation between Thr401 and Arg467 in the mutated receptor. Both modifications are likely to stabilize the active state which is in agreement with an increase in constitutive activity. In contrast, Met398^2.43^ in the model of the structure of the LHR makes two strong and one weak hydrophobic interactions in the inactive conformation and two weak hydrophobic interactions in the active conformation (Supplementary Table 2). Breaking of these interactions by the M398T mutation would destabilize the inactive conformation more than the active conformation and this is consistent with a 25-fold increase in constitutive activity compared to the wild-type receptor (Table 3).

**Table 2:** FSH receptor-activating mutations reported in the literature

Mutation	BW number	Type	Activity	References
M401T	2.43	CAM	cAMP 3.60-fold WT	[64]
T449A	3.32	CAM	cAMP 2.5-fold WT	[65]
L460R	3.43	CAM	cAMP 5-fold WT	[66]
I545T	5.46	CAM	cAMP 2.3-fold WT	[65]
I545L	5.46	CAM	cAMP 4.53-fold WT	[64]
D567N	6.30	CAM	cAMP 3-fold WT	[65]
M574I	6.37	CAM	cAMP 2.19-fold WT	[64]
A575V	6.38	CAM	cAMP 2.95-fold WT	[64]
I578L	6.41	CAM	cAMP 2.49-fold WT	[64]
T580I	6.43	CAM	cAMP 5.41-fold WT	[64]

Mutations causing increase of constitutive activity less than twofold of WT are not included

*CAM* constitutively active mutant, *BW* Ballesteros–Weinstein numbering, *WT* wild type

**Table 3:** LH receptor-activating mutations reported in the literature

Mutation	BW number	Type	Activity	References
A373V	1.46	CAM	cAMP 7.5-fold WT	[67]
M398T	2.43	CAM	cAMP 25-fold WT	[68]
L457R	3.43	CAM	cAMP 10-fold WT	[46]
I542L	5.55	CAM	cAMP 7-fold WT	[48]
D564G	6.30	CAM	cAMP 5-fold WT	[48]
A568V	6.34	CAM	cAMP 4-fold WT	[69]
M571I	6.37	Activating	–	[70]
A572V	6.38	Activating	–	[70]
I575L	6.41	CAM	cAMP 20-fold WT	[71]
T577I	6.43	Activating	–	[70]
D578G	6.44	CAM	cAMP 6.6-fold WT	[49]
D578Y	6.44	CAM	cAMP 13-fold WT	[48]
D578E	6.44	CAM	cAMP 4.3-fold WT	[49]
D578H	6.44	CAM	cAMP 14.4-fold WT	[72]
D578Q	6.44	CAM	cAMP 10.2-fold WT	[72]
C581R	6.47	CAM	cAMP 5-fold WT	[48]
N615R	7.45	CAM	cAMP 2.6-fold WT	[72]

Mutations causing increase of constitutive activity less than twofold of WT are not included

*CAM* constitutively active mutant, *BW* Ballesteros–Weinstein numbering, *WT* wild type

**Table 4:** TSH receptor-activating mutations reported in the literature

Mutation	BW number	Type	Activity	References
V421I	1.39	CAM	cAMP 2.1-fold WT	[73]
Y466A	2.56	CAM	cAMP 2.8-fold WT	[73]
I486N	ECL1	CAM	cAMP 4-fold WT	[74]
T501A	3.32	CAM	cAMP 3.4-fold WT	[73]
L512R	3.43	CAM	cAMP 3.2-fold WT	[40]
A593G	5.50	CAM	cAMP 2.13-fold WT	[15]
L629F	6.40	CAM	cAMP 2.2-fold WT	[45]
F631I	6.42	CAM	cAMP 3.7-fold WT	[75]
T632A	6.43	CAM	cAMP 9.7-fold WT	[16]
D633A	6.44	CAM	cAMP 13.6-fold WT	[16]
D633E	6.44	CAM	cAMP 3.3-fold WT	[40]
C636R	6.47	CAM	cAMP 7.7-fold WT	[76]
C636S	6.47	CAM	cAMP 5.5-fold WT	[76]
M637C	6.48	CAM	cAMP 2.4-fold WT	[73]
M637W	6.48	CAM	cAMP 4.8-fold WT	[73]
P639G	6.50	CAM	cAMP 4.9-fold WT	[76]
P639A	6.50	CAM	cAMP 4.8-fold WT	[76]
P639S	6.50	CAM	cAMP 4.8-fold WT	[76]
S641A	6.52	CAM	cAMP 3.1-fold WT	[73]
Y643F	6.54	CAM	cAMP 2.1-fold WT	[73]
L645V	6.56	CAM	cAMP 2.1-fold WT	[73]
L665F	7.40	CAM	cAMP 3-fold WT	[14]
N674D	7.49	CAM	cAMP 11.3-fold WT	[16]

Mutations causing increase of constitutive activity less than twofold of WT are not included

*CAM* constitutively active mutant, *BW* Ballesteros–Weinstein numbering, *WT* wild type

In another example, L^3.43^R mutation increases constitutive activity for the TSHR, FSHR and LHR (3.2, 5 and 10 times wild-type receptor activity, respectively, Tables 2, 3, 4). In the models, Leu^3.43^ undergoes similar interactions in the three receptors: short distance induced dipole with Asp^6.44^ in both the active and inactive conformations; long distance hydrophobic interaction with Leu^2.46^ in both conformations; short distance hydrophobic interaction with Tyr^7.53^ in the active conformations; and long distance induced dipole interactions with Asn^7.45^ and Asn^7.49^ in the active conformations (Supplementary Tables 1–3). In the L^3.43^R mutants of the GHPRs, the interactions in the active conformations between the new Arg^3.43^ and Asn^7.45^ and Asn^7.49^ are likely to be hydrogen bonds. This situation would stabilize the active conformation, increasing the constitutive activity of the three receptors as observed experimentally (Tables 2, 3, 4).

**Fig. 3:**
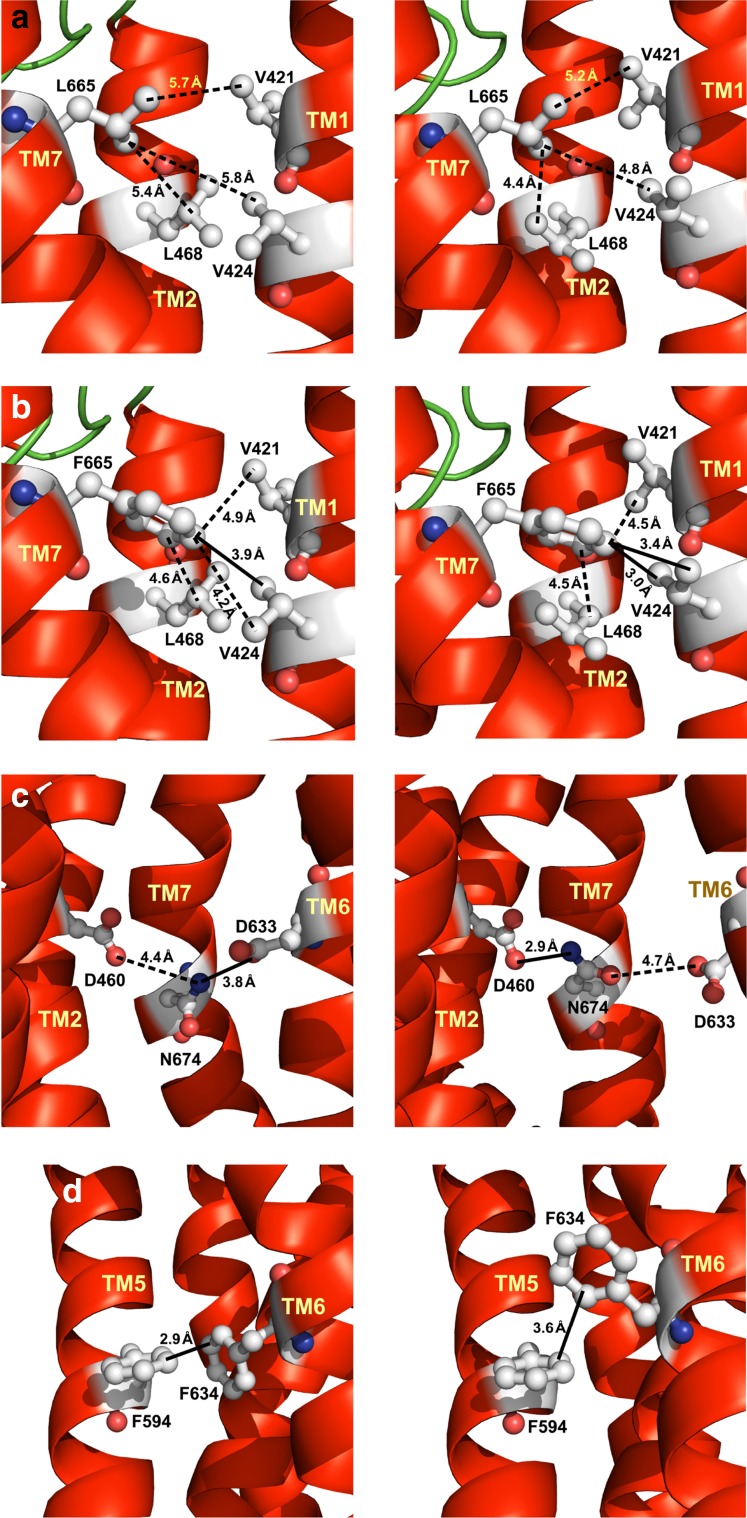
**a** Distances between thyroid-stimulating hormone receptor (TSHR) Leu665^7.40^ and Val421^1.39^ and between Val424^1.42^ and Leu468^2.58^ in the inactive conformation (*left panel*) and active conformation (*right panel*) of the TSHR transmembrane domain (TMD) wild type. **b** Distances (*dotted lines*) and interactions (*solid lines*) between TSHR Phe665^7.40^ and Val421^1.39^ and between Val424^1.42^ and Leu468^2.58^ in the inactive conformation (*left panel*) and active conformation (*right panel*) of the TSHR TMD L665F mutant. **c** Distances (*dotted lines*) and interactions (*solid lines*) of TSHR Asn674^7.49^ with Asp460 and Asp633^6.44^ in the inactive conformation (*left panel*) and active conformation (*right panel*) and **d** between Phe594^5.51^ and Phe634^6.54^ in the inactive conformation (*left panel*) and active conformation (*right panel*) of the TSHR TMD. Reproduced with permission from copyright holder RSR Ltd

TSHR mutation L665^7.40^F increases receptor constitutive activity threefold compared to wild type (Table 4) in COS-7 cells transfected with the wild-type and mutated TSHR. Cell surface expression of the L665^7.40^F mutant is 97% of that of the wild-type TSHR. Stimulation of the mutant by 100 mU/mL TSH produced a slightly higher cAMP accumulation (65.2 nM) than that of the wild-type TSHR (52.7 nM) [14]. As indicated in Supplementary Table 3, the TSHR residue Leu665 is not involved in interactions with the other helices or loops in any of the models of the two conformations of the TSHR TMD, active and inactive. In the inactive conformation, the side chain of Leu665^7.40^ is 5.4, 5.7 and 5.8 Å away from the side chains of Leu468^2.58^, Val421^1.39^ and Val424^1.42^, respectively (Fig. 3a). In the active conformations, Leu665^7.40^ is located at shorter but still non-interacting distances, 4.4, 5.2 and 4.8 Å away from the side chains of Leu468^2.58^, Val421^1.39^ and Val424^1.42^, respectively (Fig. 3a). Introduction of a longer side chain at position 665 by the L665F mutation is likely to generate hydrophobic interactions with the side chains of some of the above-mentioned residues. In particular, the mutated TSHR Phe665 is likely to interact with Val424 at ~3.9 Å in the inactive conformation (Fig. 3b) and to form a double hydrophobic interaction at ~3.0 and ~3.4 Å in the active conformation (Fig. 3b). This implies that the mutant stabilizes the active more than the inactive conformation, consistent with a higher constitutive activity of the mutant with respect to the wild-type receptor. Jaeschke et al. [14] also studied the L665^7.40^F mutant with their model of the TSHR TMD based on the templates of inactive, partially active and fully active GPCRs. Their model predicted an interaction between Leu665 and Val421 and suggested a steric repulsion of the longer side chain of Phe655. This observation contrasts with the interpretation from our models that suggests additional interactions that occur in the active conformation of L655F mutant formed by the long side chain of Phe655. Jaeschke et al.’s [14] prediction was supported by comparing the structures of inactive and partially active A2A adenosine receptor, showing an increased distance between the residues equivalent to Leu665 and Val421 in the partially active conformation. In contrast, when comparing inactive with fully active conformations, the situation reverses. The distance between the residues equivalent to Leu665 and Val421 in the inactive rhodopsin (PB-Id: 1U19) is 3.8 Å between the side chains and 9.0 Å between the alpha carbon atoms (Cα), whereas in the fully active conformation (PDB-Id: 3PQR) those distances are 3.2 and 8.8 Å, respectively. In the case of the β2-adrenoceptor (inactive: 2RH1, fully active: 3SN6), the distances in the inactive state are 3.9 Å between side chains and 10.0 Å between Cαs, whereas in the fully active state the distances are 3.9 and 9.3 Å, respectively. For the inactive M2 muscarinic receptor (3UON), the distances are 4.1 and 9.6 Å, whereas for the fully active receptor (4MQS) they are 3.5 and 8.5 Å, respectively. These observations suggest that helices TM1 and TM7 as well as Leu655 and Val421 approach each other during activation. Furthermore, the effects of substitutions with small amino acids in mutations V421A or L665V resulting in TSHR variants exhibiting ligand-induced cAMP levels similar to or below the levels of the wild-type receptor were inconsistent with steric repulsion proposed by Jaeschke et al. [14] in their model. In contrast, in our models the proposed additional interactions produced by V421 and L665 in the active state of the receptor would be weakened by substitutions with the residues with smaller side chains.

### Silencing and inactivating mutations in the GPHR TMDs

Tables 5, 6 and 7 show a list of silencing and/or inactivating mutations taken from the literature, whereas Supplementary Tables 4–6 list the main interactions in which those mutant residues are involved. For example, FSH-induced cyclic AMP production is abolished by the FSHR mutation A575^6.38^V (Supplementary Table 4). In both models of the active and inactive conformations of the FSHR TMD, Tyr553^5.62^ is exposed to the membrane. In the active conformation, the hydroxyl group of Tyr553 is located in the area that contacts the polar heads of lipids at the inner side of the membrane, making favourable interactions. In the inactive conformation, the hydroxyl group of Tyr553 is located in an area that contacts the aliphatic tails of the lipids in the membrane which introduces instability by placing the polar tyrosine hydroxyl group in a hydrophobic environment. In the FSHR TMD A575V mutant, the side chain of Val575 covers the hydroxyl group of Tyr553, in the inactive conformation, and protects it from contacting the membrane, which increases the stability of the inactive conformation of the TMD. In the active conformation, Tyr553 is not affected by the A575V mutation. The increased stability of the inactive conformation is consistent with the experimentally demonstrated reduced activation of the mutated receptor.

**Table 5:** FSH receptor-inactivating/silencing mutations reported in the literature

Mutation	BW number	Type^a^	Activity	References
I418S	2.60	Inactivating	FSH-induced cAMP abolished	[77]
A419T	2.61	Inactivating	FSH-induced cAMP abolished	[78]
P519T	ECL2	Inactivating	FSH-induced cAMP abolished	[65]
R573C	6.36	Inactivating	FSH-induced max. cAMP 30% WT	[79]
A575V	6.38	Inactivating	FSH-induced cAMP abolished	[80]
P587H	6.50	Inactivating	FSH-induced cAMP abolished	[65]
L601V	ECL3	Inactivating	FSH-induced max. cAMP 20% WT	[81]

*BW* Ballesteros–Weinstein numbering, *WT* wild type

^a^Mutation causing decrease of the receptor constitutive activity is defined as silencing. Mutation causing decrease of hormone-induced cyclic AMP activity is defined as inactivating

**Table 6:** LH receptor inactivating/silencing mutations reported in the literature

Mutation	BW number	Type^a^	Activity	References
I374T	1.47	Inactivating	–	[82]
T392I	ICL1	Inactivating	–	[82]
C543R	5.55	Inactivating	LH-induced cAMP abolished	[83]
A593P	6.59	Inactivating	LH-induced cAMP abolished	[84]
S616Y	7.46	Inactivating	LH-induced EC_50_ 20-fold WT	[85]
I625K	7.55	Inactivating	LH-induced EC_50_ 20-fold WT	[85]

*BW* Ballesteros–Weinstein numbering, *WT* wild type

^a^Mutation causing decrease of the receptor constitutive activity is defined as silencing. Mutation causing decrease of hormone-induced cyclic AMP activity is defined as inactivating

**Table 7:** TSH receptor-inactivating/silencing mutations reported in the literature

Mutation	BW number	Type^a^	Activity	References
V424I	1.42	Silencing	cAMP 0.3-fold WT	[86]
D460A	2.50	Silencing	cAMP 0.28-fold WT	[16]
D460N	2.50	Silencing	cAMP 0.18-fold WT	[16]
L467V	2.57	Silencing	cAMP 0.1-fold WT	[86]
W488R	ECL1	Inactivating	TSH binding abolished	[87]
V502A	3.33	Silencing	cAMP 0.0-fold WT	[86]
M527T	ICL2	Inactivating	TSH induced max. cAMP 30% WT	[87]
Y582A	5.39	Silencing	cAMP 0.2-fold WT	[86]
Y582F	5.39	Silencing	cAMP 0.4-fold WT	[86]
A593P	5.50	Silencing	cAMP 0.19-fold WT	[15]
A593V	5.50	Silencing	cAMP 0.33-fold WT	[15]
F594I	5.51	Silencing	cAMP 0.13-fold WT	[15]
R625A	6.36	Silencing	cAMP 0.11-fold WT	[16]
F634I	6.45	Silencing	cAMP 0.13-fold WT	[15]
A638V	6.49	Inactivating	TSH induced EC_50_ 7.85-fold WT	[15]
F642I	6.53	Inactivating	TSH induced EC_50_ 8.21-fold WT	[15]
Y643A	6.54	Silencing	cAMP 0.2-fold WT	[86]
A644V	6.55	Inactivating	TSH induced EC_50_ 8.88-fold WT	[15]
L665V	7.40	Silencing	cAMP 0.3-fold WT	[86]

Mutations causing decrease of constitutive activity above 0.5-fold of WT are not included

*BW* Ballesteros–Weinstein numbering, *WT* wild type

^a^Mutation causing decrease of the receptor constitutive activity is defined as silencing. Mutation causing decrease of hormone-induced cyclic AMP activity is defined as inactivating

In another example, LH-induced cyclic AMP production by LHR is abolished by the LHR A593^6.59^P mutation (Table 6). In the model of the inactive conformation of the LHR TMD wild type, the beta carbon atom of Ala593^6.59^ forms a hydrophobic interaction at 3.4 Å with the delta 1 carbon atom of Ile528^5.40^ (Supplementary Table 5). No interaction is observed in the model of the active conformation, as the distance between these two residues is 4.9 Å. In the inactive conformation of the LHR TMD A593^6.59^P mutant, Pro593^6.59^ is likely to have additional hydrophobic interaction with Ile528^5.40^. In particular, the gamma carbon atom of Pro593 is likely to interact with the delta 1 carbon atom of Ile528 at 3.4 Å, and the beta carbon atom of Pro593 is likely to interact with the delta 1 and the gamma 1 carbon atoms of Ile528 at 3.4 and 4.0 Å, respectively. The extra interactions between Pro593 in TM6 and Ile528 in TM5 in the inactive conformation may block the rotation and displacement of TM6 needed for LHR activation, making the A593P mutant unable to be activated by LH, as observed experimentally (Table 6).

TSHR F594I and F634I single mutants have been reported to cause silencing of the constitutive activity of the receptor to ~13% of the wild type (Table 7). The model of the inactive conformation of the TSHR TMD predicts an aromatic interaction between Phe594^5.51^ and Phe634^6.54^ at 2.9 Å (Supplementary Table 6, Fig. 3d). Substitution of any phenylalanine with isoleucine is likely to maintain a hydrophobic interaction due to this short distance. In addition, the model of the active conformation of the TSHR TMD predicts an aromatic interaction between the two phenylalanines at 3.6 Å (Supplementary Table 6, Fig. 3d). Substitution of any phenylalanine with a residue with a shorter side chain, for example isoleucine, is likely to eliminate the interaction. In the active wild type receptor, the distance between the interacting phenylalanines is longer than in the inactive wild-type receptor. Consequently, substitution of any of these two phenylalanines with isoleucine is likely to have little effect on the inactive conformation of the receptor, while destabilizing the active conformation, consistent with the reduction of constitutive activity observed for both receptor mutants.

In a final example, the model of the inactive conformation of the TSHR TMD predicts that Asn674^7.49^ makes a polar interaction or weak hydrogen bond, with Asp633^6.44^ (at 3.8 Å) (Supplementary Table 3, Fig. 3c). TSHR mutant D633A increases the constitutive activity by 13.6-fold compared to the wild type (Table 4), even though the cell surface expression of the mutant is only 26% of that of the wild-type TSHR. The TSHR D633A mutant also shows an increase in maximum cAMP accumulation of 50% compared to wild type when stimulated by 10 mIU/ml bTSH. This increase in constitutive activity of the mutant is due to a destabilization of the inactive conformation of the receptor by breaking the polar interaction. In contrast, in the model of the active conformation of the TSHR TMD, Asn674^7.49^ makes a hydrogen bond with Asp460^2.50^ (Supplementary Table 6, Fig. 3c). The TSHR mutant D460A reduces the constitutive activity to 28% of the wild type (Table 7), due to a destabilization of the active conformation of the receptor by breaking the hydrogen bond.

In conclusion, to study activation of the GPHR TMDs, we built comparative models of the inactive and fully active conformations of their TMDs based on three GPCRs for which the crystal structures of both the inactive and fully active conformations were available. Most GPCR-conserved helix distortions are observed in our models of both the active and inactive GPHR TMD conformations. In addition, most GPCR TMD-conserved motifs are observed in the amino acid sequences of the GPHR TMDs. Some of these conserved helix distortions and motifs have not been described previously for GPHRs. Furthermore, GPCR transmembrane helix displacements and rotations observed during receptor activation are also observed when comparing the inactive and active conformations of the GPHR TMDs. In addition, our study also suggests GPHR-specific hydrophobic interactions between residues at positions 3.30 and 4.58. A detailed analysis of the active and inactive conformations of GPHR TMD structures produced by comparative modelling has not been carried out before. Furthermore, our TMD models were used to study the effects of previously reported GPHR TMD mutations on the molecular interactions and activity of the GPHRs which had not been previously explained at the structural level. We propose that the conformational modifications of the transmembrane helices and rearrangements of important interactions during activation of the GPHR TMD are similar to those previously reported for GPCR activation. During activation of GPHRs, an ionic lock is broken and important hydrogen bonds and hydrophobic interactions are rearranged. All these modifications open the G-protein binding site at the cytoplasmic end of their TMD structures.

## Electronic supplementary material

Below is the link to the electronic supplementary material.

Supplementary material 1 (DOC 1510 kb)
