# Supplementary material for: Structure and activation of the TSH receptor transmembrane domain
Source: Auto Immun Highlights. 2016 Dec 5;8(1):2. doi: 10.1007/s13317-016-0090-1 (PMC5136658; doi:10.1007/s13317-016-0090-1)
Supplement: Supplementary file 1 — Supplementary material 1 (DOC 1510 kb) [file 13317_2016_90_MOESM1_ESM.doc]

**SUPPLEMENTARY MATERIAL**

# **Structure and Activation of the TSH Receptor Transmembrane Domain**

**Autoimmunity Highlights**

Ricardo Núñez Miguel, Jane Sanders, Jadwiga Furmaniak and Bernard Rees Smith

FIRS Laboratories, RSR Ltd, Parc Ty Glas, Llanishen, Cardiff, CF14 5DU, UK

Corresponding author: Dr Bernard Rees Smith, FIRS Laboratories, RSR Ltd, Parc Ty Glas, Llanishen, Cardiff, CF14 5DU, UK. Tel: +44 29 2076 5550; Fax: +44 29 2076 4575; email: [firs@rsrltd.eclipse.co.uk](mailto:firs@rsrltd.eclipse.co.uk)

**Supplementary Figures**

**Supplementary Figure 1**

Amino acid sequence alignment used in the comparative modelling of the structure of the inactive thyroid stimulating hormone receptor (TSHR) transmembrane domain (TMD_I). Homologous proteins are named by their Protein Data Bank IDs (Table 1). Dark green rectangles denote segments of residues and number of residues that have been deleted for this study from the coordinates files. Light green rectangles indicates residues that were not present in the original coordinate files. N- or C-terminal residues deleted or not included in the original coordinate files are not marked. Alignment formatted with Discovery Studio 3.5. Reproduced with permission from copyright holder RSR Ltd.


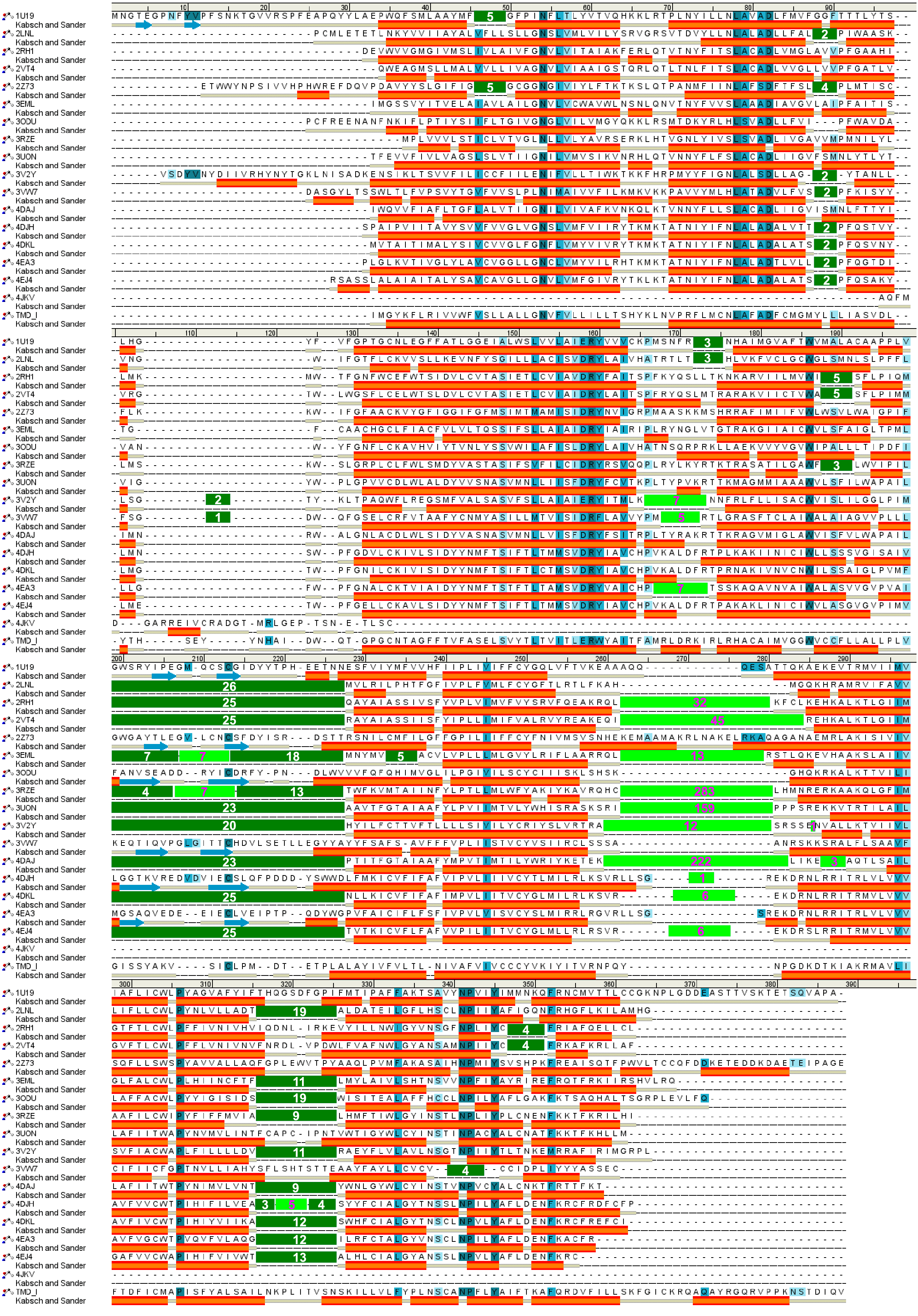


**Supplementary Figure 2**

Amino acid sequence of the thyroid stimulating hormone receptor (TSHR) transmembrane domain. TSHR residues showing helix structure are shown in bold and underlined. Transmembrane helices (TM) are marked and numbered TM1 to TM7 as well as helix 8 (H8). Extracellular (ECL) and intracellular (ICL) loops are marked and numbered 1 to 3. TSHR sequence numbering is shown on top of the amino acid sequence. Ballesteros-Weinstein numbering is shown under the amino acid sequence. In the Ballesteros-Weinstein numbering, residue 50 of each helix is the most conserve residue of the helix in the GPCR superfamily. Reproduced with permission from copyright holder RSR Ltd.


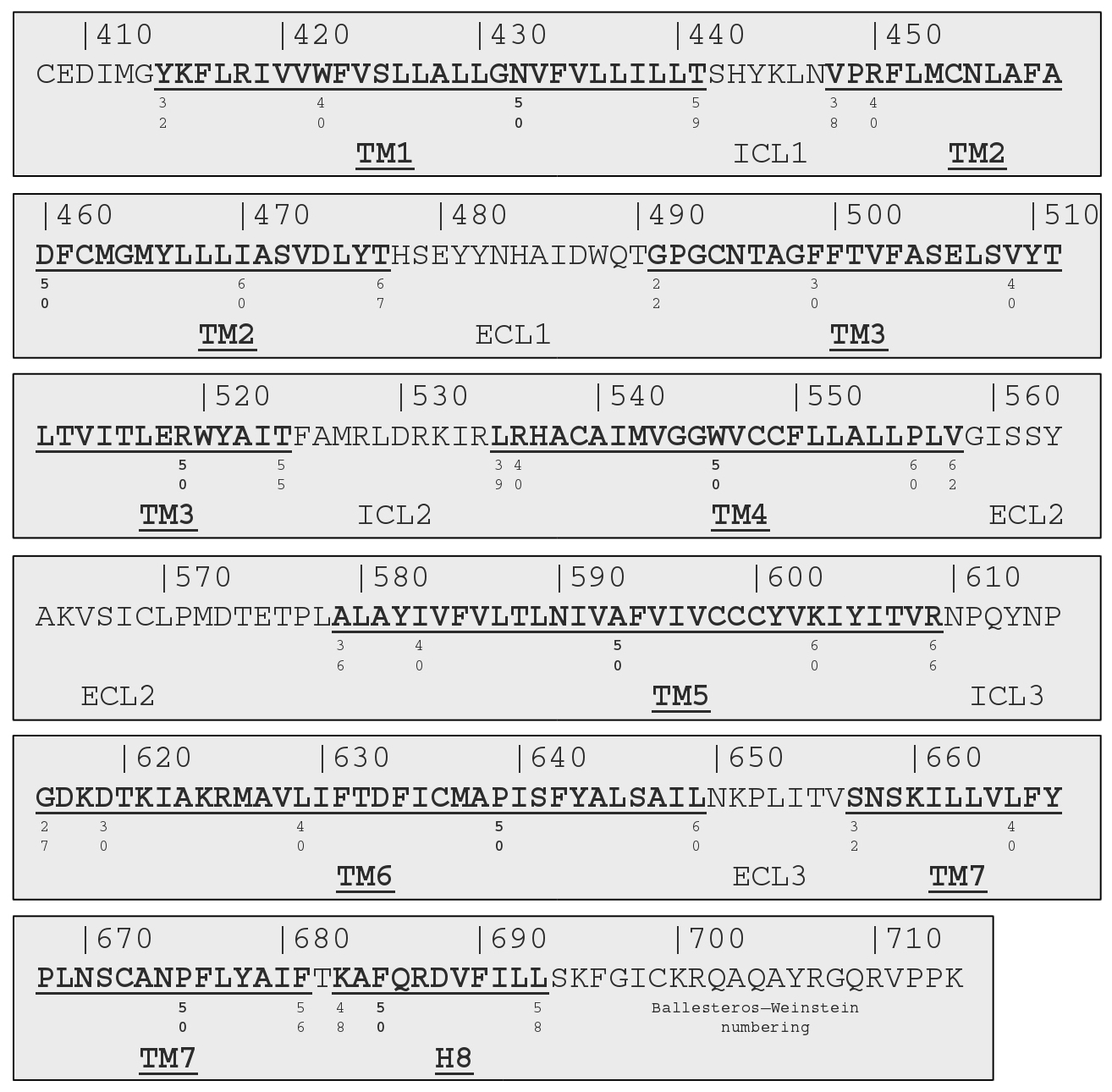


**Supplementary Figure 3**

Amino acid sequence alignment used in the comparative modelling of the structure of the inactive thyroid stimulating hormone receptor (TSHR) transmembrane domain (TMD_In). Homologous proteins are named by their Protein Data Bank IDs. Dark green rectangles denote segments of residues and the number of residues that have been deleted for this study from the coordinates files. Light green rectangles indicates residues that were not present in the coordinate files. N- or C-terminal residues deleted or not included in the coordinate files are not marked. Alignment formatted with Discovery Studio 3.5. Reproduced with permission from copyright holder RSR Ltd.


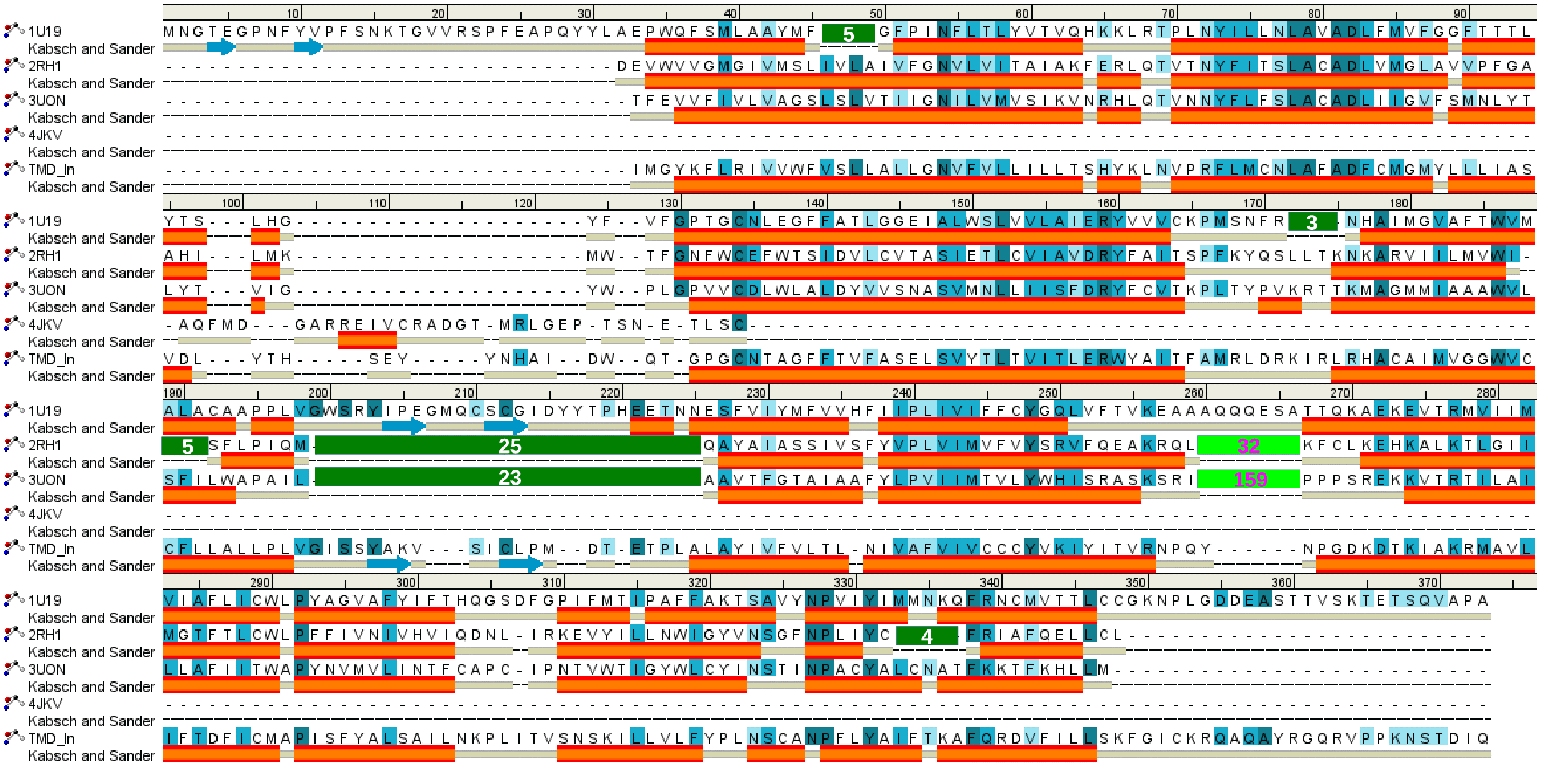


**Supplementary Figure 4**

Amino acid sequence alignment used in the comparative modelling of the structure of the active thyroid stimulating hormone receptor (TSHR) transmembrane domain (TMD_Ac). Homologous proteins are named by their Protein Data Bank IDs. Dark green rectangles denote segments of residues and the number of residues that have been deleted for this study from the coordinates files. Light green rectangles indicates residues that were not present in the coordinate file. N- or C-terminal residues deleted or not included in the coordinate files are not marked. Alignment formatted with Discovery Studio 3.5. Reproduced with permission from copyright holder RSR Ltd.


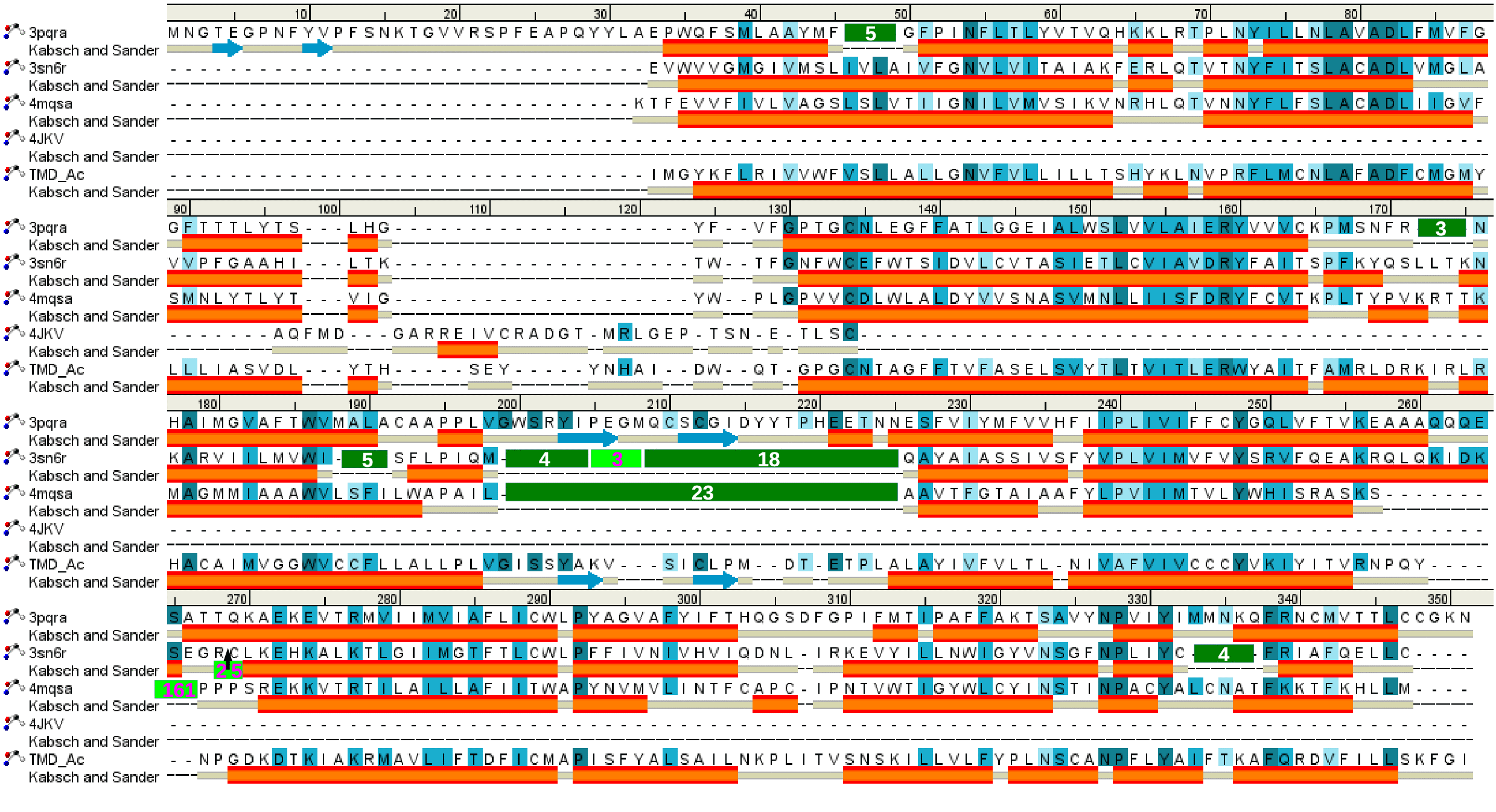


**Supplementary Tables**

**Supplementary Table 1**

FSH receptor residues involved in activating mutations and their main interactions.

| Mutation | BW number | Interactions | | | |
| --- | --- | --- | --- | --- | --- |
|  |  | Conformation | Type | Residue (BW) | Distance |
| M401T | 2.43 | Inactive | Hydrophobic | I463(3.46) | 3.6 Å |
|  |  | Active | Induce dipole | R467(3.50) | 3.4 Å |
| T449A | 3.32 | Inactive | Hydrophobic  Induce dipole | L415(2.57)  C517(ECL2) | 3.0 Å  3.8 Å |
|  |  | Active | Hydrophobic | L415(2.57) | 2.9 Å |
| L460R | 3.43 | Inactive | Induce dipole  Hydrophobic | D581(6.44)  L577(6.40) | 3.0 Å  2.9 Å |
|  |  | Active | Hydrophobic  Induce dipole  Induce dipole  Induce dipole  Hydrophobic | L404(2.46)  D581(6.44)  N618(7.45)  N622(7.49)  Y626(7.53) | 3.6 Å  3.1 Å  3.7 Å  3.8 Å  3.0 Å |
| I545T/L | 5.46 | Inactive | Hydrophobic  Induce dipole  Hydrophobic | T461(3.44)  T464(3.47)  I578(6.41) | 3.9 Å  3.3 Å  3.9 Å |
|  |  | Active | Hydrophobic  Polar  Induce dipole | T461(3.44)  T464(3.47)  D581(6.44) | 3.9 Å  3.8 Å  3.8 Å |
| D567N | 6.30 | Inactive | Hydrophobic  HB (backbone-backbone)  Induce dipole | L477(ICL2)  S565(ICL3)  I561(ICL3) | 3.3 Å  3.4 Å  3.3 Å |
|  |  | Active | HB (backbone-side chain)  Polar  HB (side chain-backbone) | Y553(5.62)  V556(5.65)  R557(5.66) | 3.0 Å  3.8 Å  3.4 Å |
| M574I | 6.37 | Inactive | Hydrophobic  Induce dipole | I463(3.46)  Y549(5.58) | 2.9 Å  3.3 Å |
|  |  | Active | Hydrophobic | Y549(5.58) | 3.4 Å |
| A575V | 6.38 | Inactive | Induce dipole | Y553(5.62) | 2.8 Å |
|  |  | Active | - | - | - |
| I578L | 6.41 | Inactive | Hydrophobic | I545(5.54) | 3.9 Å |
|  |  | Active | - | - | - |
| T580I | 6.43 | Inactive | Induce dipole/hydrophobic  hydrophobic | N618(7.45)  A621(7.48) | 3.0 Å  3.9 Å |
|  |  | Active | Polar  Induce dipole/hydrophobic | N618(7.45)  L625(7.52) | 3.9 Å  3.0 Å |

BW = Ballesteros-Weinstein numbering

HB = hydrogen bonds (s.c. = side chain; b.b. = backbone)

References in Table 2A

**Supplementary Table 2**

LH receptor residues involved in activating mutations and their main interactions.

| Mutation | BW number | Interactions | | | |
| --- | --- | --- | --- | --- | --- |
|  |  | Conformation | Type | Residue (BW) | Distance |
| A373V | 1.46 | Inactive | Hydrophobic | C617(7.47) | 3.7 Å |
|  |  | Active | Hydrophobic | C617(7.47) | 3.4 Å |
| M398T | 2.43 | Inactive | Hydrophobic  Hydrophobic/Polar  Hydrophobic | V380(1.53)  Y623(7.53)  F630(8.50) | 3.9 Å  3.3 Å  3.0 Å |
|  |  | Active | Hydrophobic  Hydrophobic | I460(3.46)  F630(8.50) | 3.9 Å  3.9 Å |
| L457R | 3.43 | Inactive | Induced dipole/hydrophobic | D578(6.44) | 3.0 Å |
|  |  | Active | Hydrophobic  Induced dipole/hydrophobic  Induced dipole  Induced dipole  Induced dipole/hydrophobic | L401(2.46)  D578(6.44)  N615(7.45)  N619(7.49)  Y623(7.53) | 3.7 Å  2.8 Å  3.6 Å  3.9 Å  3.1 Å |
| I542L | 5.55 | Inactive | Hydrophobic  Hydrophobic/polar  Hydrophobic | T458(3.44)  T461(3.47)  I575(6.41) | 3.6 Å  3.6 Å  3.6 Å |
|  |  | Active | Hydrophobic  Induced dipole/hydrophobic | T458(3.44)  T461(3.47) | 3.7 Å  3.2 Å |
| D564G | 6.30 | Inactive | - | - | - |
|  |  | Active | - | - | - |
| A568V | 6.34 | Inactive | HB (backbone-side chain) | Y546(5.58) | 2.6 Å |
|  |  | Active | Hydrophobic | Y550(5.62) | 3.0 Å |
| M571I | 6.37 | Inactive | Hydrophobic  Hydrophobic  Induced dipole | P394(2.39)  I460(3.46)  Y546(5.58) | 3.7 Å  3.2 Å  3.3 Å |
|  |  | Active | Induced dipole | Y550(5.62) | 3.4 Å |
| A572V | 6.38 | Inactive | Polar  Induced dipole | Y546(5.58)  Y550(5.62) | 3.7 Å  2.9 Å |
|  |  | Active | - | - | - |
| I575L | 6.41 | Inactive | Hydrophobic | I542(5.54) | 3.6 Å |
|  |  | Active | - | - | - |
| T577I | 6.43 | Inactive | Induced dipole/hydrophobic | N615(7.45) | 2.9 Å |
|  |  | Active | Induced dipole  Hydrophobic  Hydrophobic | N615(7.45)  A618(7.48)  L622(7.52) | 3.4 Å  3.8 Å  3.2 Å |
| D578G/Y/  /E/H/Q | 6.44 | Inactive | Induced dipole/hydrophobic  HB (side chain-side chain) | L457(3.43)  N615(7.45) | 3.0 Å  2.9 Å |
|  |  | Active | Induced dipole/hydrophobic  Polar  Polar | L457(3.43)  N615(7.45)  Y623(7.53) | 2.8 Å  3.7 Å  3.6 Å |
| C581R | 6.47 | Inactive | Hydrophobic  Induced dipole  HB (side chain-side chain) | F611(7.41)  I614(7.44)  N615(7.45) | 2.9 Å  3.8 Å  3.0 Å |
|  |  | Active | Hydrophobic  Polar  HB (side chain-side chain) | F611(7.41)  Y612(7.42)  N615(7.45) | 3.1 Å  3.6 Å  3.5 Å |
| N615R | 7.45 | Inactive | HB (side chain-backbone)  2HB (s.c.-b.b., s.c.-s.c.)  HB (side chain-side chain) | T577(6.43)  D578(6.44)  C581(6.47) | 3.0 Å  2.9 Å  3.0 Å |
|  |  | Active | Induced dipole  Induced dipole  Polar  HB (side chain-side chain) | L457(3.43)  T577(6.43)  D578(6.44)  C581(6.47) | 3.6 Å  3.4 Å  3.7 Å  3.5 Å |

BW = Ballesteros-Weinstein numbering

HB = hydrogen bonds (s.c. = side chain; b.b. = backbone)

References in Table 2B

**Supplementary Table 3**

TSH receptor residues involved in activating mutations and their main interactions.

| Mutation | BW number | Interactions | | | |
| --- | --- | --- | --- | --- | --- |
|  |  | Conformation | Type | Residue (BW) | Distance |
| V421I | 1.39 | Inactive | Induce dipole/hydrophobic  Induce dipole/hydrophobic | L468(2.58)  S472(2.62) | 3.5 Å  3.1 Å |
|  |  | Active | Hydrophobic | S472(2.62) | 3.2 Å |
| Y466A | 2.56 | Inactive | - | - | - |
|  |  | Active | - | - | - |
| I486N | ECL1 | Inactive | Hydrophobic  Hydrophobic  Hydrophobic | G491(3.22)  C494(3.25)  K565(ECL2) | 3.4 Å  3.7 Å  3.0 Å |
|  |  | Active | Induced dipole  Hydrophobic  Induced dipole  Hydrophobic  Hydrophobic | D474(2.64)  L475(2.65)  V566(ECL2)  S567(ECL2)  I661(7.36) | 3.7 Å  3.4 Å  3.8 Å  2.9 Å  3.4 Å |
| T501A | 3.32 | Inactive | Hydrophobic  Hydrophobic  Polar | M463(2.53)  L467(2.57)  C569(ECL2) | 3.9 Å  3.5 Å  3.7 Å |
|  |  | Active | Hydrophobic  Polar | L467(2.57)  C569(ECL2) | 2.9 Å  3.6 Å |
| L512R | 3.43 | Inactive | Hydrophobic  Hydrophobic  Induced dipole | L456(2.46)  L629(6.40)  D633(6.44) | 3.9 Å  3.2 Å  3.0 Å |
|  |  | Active | Hydrophobic  Induced dipole  Induced dipole  Induced dipole  Hydrophobic | L456(2.46)  D633(6.44)  N670(7.45)  N674(7.49)  Y678(7.53) | 3.7 Å  3.1 Å  3.7 Å  3.8 Å  3.4 Å |
| A593G | 5.50 | Inactive | Hydrophobic | V509(3.40) | 3.7 Å |
|  |  | Active | Induced dipole | T513(3.44) | 3.4 Å |
| L629F | 6.40 | Inactive | Hydrophobic  Hydrophobic  Induced dipole | L456(2.46)  L512(3.43)  Y678(7.53) | 3.1 Å  3.2 Å  3.9 Å |
|  |  | Active | Induced dipole  Induced dipole  Hydrophobic  Hydrophobic | R519(3.50)  Y601(5.58)  L677(7.52)  Y678(7.53) | 2.9 Å  3.7 Å  3.9 Å  3.9 Å |
| F631I | 6.42 | Inactive | - | - | - |
|  |  | Active | - | - | - |
| T632A | 6.43 | Inactive | HB (side chain-side chain) | N670(7.45) | 3.3 Å |
|  |  | Active | Polar  Hydrophobic  Hydrophobic | N670(7.45)  A673(7.48)  L677(7.52) | 3.9 Å  3.8 Å  2.8 Å |
| D633A/E | 6.44 | Inactive | Induced dipole  HB (side chain-side chain)  Polar | L512(3.43)  N670(7.45)  N674(7.49) | 3.0 Å  2.9 Å  3.8 Å |
|  |  | Active | Induced dipole  Polar | L512(3.43)  Y678(7.53) | 3.1 Å  3.9 Å |
| C636R/S | 6.47 | Inactive | Hydrophobic  HB (side chain-side chain) | F666(7.41)  N670(7.45) | 2.9 Å  3.2 Å |
|  |  | Active | HB (side chain-backbone)  Polar | F666(7.41)  N670(7.45) | 3.6 Å  3.5 Å |
| M637C/W | 6.48 | Inactive | Induce dipole/hydrophobic | Y667(7.42) | 3.3 Å |
|  |  | Active | Induced dipole  hydrophobic | V509(3.40)  Y667(7.42) | 3.1 Å  3.1 Å |
| P639G/A/S | 6.50 | Inactive | Hydrophobic | L663(7.38) | 3.0 Å |
|  |  | Active | Hydrophobic | L663(7.38) | 2.7 Å |
| S641A | 6.52 | Inactive | - | - | - |
|  |  | Active | Induced dipole  HB (side chain-side chain) | L587(5.44)  N590(5.47) | 3.8 Å  2.9 Å |
| Y643F | 6.54 | Inactive | HB (backbone-side chain)  Hydrophobic | K660(7.35)  L663(7.38) | 3.3 Å  3.0 Å |
|  |  | Active | Hydrophobic | L663(7.38) | 2.9 Å |
| L645V | 6.56 | Inactive | Hydrophobic | L587(5.44) | 3.6 Å |
|  |  | Active | Hydrophobic | L587(5.44) | 3.8 Å |
| L665F | 7.40 | Inactive | - | - | - |
|  |  | Active | - | - | - |
| N674D | 7.49 | Inactive | Induced dipole  Induce dipole  Induce dipole/hydrophobic  Polar | L456(2.46)  D460(2.50)  L629(6.40)  D633(6.44) | 3.6 Å  3.4 Å  2.9 Å  3.8 Å |
|  |  | Active | Induce dipole/hydrophobic  HB (side chain-side chain)  HB (side chain-side chain) | L456(2.46)  D460(2.50)  D460(2.50) | 3.4 Å  2.9 Å  3.2 Å |

BW = Ballesteros-Weinstein numbering

HB = hydrogen bonds (s.c. = side chain; b.b. = backbone)

References in Table 2C

**Supplementary Table 4**

FSH receptor residues involved in inactivating/silencing mutations and their main interactions.

| Mutation | BW number | Interactions | | | |
| --- | --- | --- | --- | --- | --- |
|  |  | Conformation | Type | Residue (BW) | Distance |
| I418S | 2.60 | Inactive | Hydrophobic | W436(ECL1) | 3.5 Å |
|  |  | Active | Hydrophobic  Hydrophobic | W436(ECL1)  I516(ECL2) | 3.5 Å  3.8 Å |
| A419T | 2.61 | Inactive | Polar | S515(ECL2) | 3.4 Å |
|  |  | Active | Polar  Induce dipole/hydrophobic | S515(ECL2)  Ile516(ECL2) | 3.5 Å  3.7 Å |
| P519T | ECL2 | Inactive | - | - | - |
|  |  | Active | Hydrophobic | V450(3.33) | 3.9 Å |
| R573C | 6.36 | Inactive | Induce dipole  Hydrophobic  HB (side chain-side chain)  induced dipole | L625(7.52)  F629(7.56)  T630(7.57)  F633(8.50) | 3.2 Å  3.6 Å  2.7 Å  3.3 Å |
|  |  | Active | Hydrophobic | F629(7.56) | 3.6 Å |
| A575V | 6.38 | Inactive | Induce dipole | Y553(5.62) | 2.8 Å |
|  |  | Active | - | - | - |
| P587H | 6.50 | Inactive | Induce dipole/hydrophobic | L611(7.38) | 2.8 Å |
|  |  | Active | Induce dipole/hydrophobic | L611(7.38) | 3.1 Å |
| L601V | ECL3 | Inactive | - | - | - |
|  |  | Active | - | - | - |

BW = Ballesteros-Weinstein numbering

HB = hydrogen bonds (s.c. = side chain; b.b. = backbone)

References in Table 3A

**Supplementary Table 5**

LH receptor residues involved in inactivating/silencing mutations and their main interactions.

| Mutation | BW number | Interactions | | | |
| --- | --- | --- | --- | --- | --- |
|  |  | Conformation | Type | Residue (BW) | Distance |
| I374T | 1.47 | Inactive | Induce dipole/hydrophobic  Induce dipole | F406(2.51)  L410(2.55) | 3.1 Å  2.9 Å |
|  |  | Active | Induce dipole/hydrophobic  Hydrophobic  Induce dipole | F406(2.51)  G409(2.54)  L410(2.55) | 3.8 Å  3.6 Å  3.7 Å |
| T392I | ICL1 | Inactive | HB (side chain-backbone)  Polar  HB (side chain-backbone)  Induce dipole/hydrophobic | V393(2.38)  P394(2.39)  R395(2.40)  F396(2.41) | 3.4 Å  3.5 Å  3.0 Å  3.0 Å |
|  |  | Active | Polar/hydrophobic  Hydrophobic  HB (side chain-backbone)  Induce dipole/polar | V393(2.38)  P394(2.39)  R395(2.40)  F396(2.41) | 2.4 Å  3.5 Å  3.5 Å  3.2 Å |
| C543R | 5.55 | Inactive | - | - | - |
|  |  | Active | - | - | - |
| A593P | 6.59 | Inactive | Hydrophobic  Induced dipole | I528(5.40)  V596(ECL3) | 3.4 Å  3.7 Å |
|  |  | Active | - | - | - |
| S616Y | 7.46 | Inactive | Polar  HB (side chain-side chain)  Induced dipole | N377(1.50)  D405(2.50)  M408(2.53) | 3.8 Å  3.2 Å  3.5 Å |
|  |  | Active | Polar  HB (side chain-side chain)  HB (side chain-side chain) | N377(1.50)  D405(2.50)  M408(2.53) | 3.6 Å  3.0 Å  2.9 Å |
| I625K | 7.55 | Inactive | - | - | - |
|  |  | Active | Polar/Induce dipole | Q631(8.51) | 2.9 Å |

BW = Ballesteros-Weinstein numbering

HB = hydrogen bonds (s.c. = side chain; b.b. = backbone)

References in Table 3B

**Supplementary Table 6**

TSH receptor residues involved in inactivating/silencing mutations and their main interactions.

| Mutation | BW number | Interactions | | | |
| --- | --- | --- | --- | --- | --- |
|  |  | Conformation | Type | Residue (BW) | Distance |
| V424I | 1.42 | Inactive | Hydrophobic  Hydrophobic  HB (backbone-side chain) | L468(2.58)  L669(7.44)  C672(7.47) | 3.0 Å  3.9 Å  3.8 Å |
|  |  | Active | - | - | - |
| D460A/N | 2.50 | Inactive | Polar  HB (side chain-side chain) | N432(1.50)  S671(7.46) | 3.7 Å  3.0 Å |
|  |  | Active | HB (backbone-side chain)  HB (backbone-side chain)  HB (side chain-side chain)  HB (side chain-side chain)  HB (side chain-side chain) | N432(1.50)  S508(3.39)  S671(7.46)  N674(7.49)  N674(7.49) | 3.4 Å  3.4 Å  3.0 Å  2.9 Å  3.2 Å |
| L467V | 2.57 | Inactive | Hydrophobic | T501(3.32) | 3.5 Å |
|  |  | Active | Hydrophobic | T501(3.32) | 2.9 Å |
| W488R | ECL1 | Inactive | Hydrophobic  Polar (HB)  Hydrophobic  Induced dipole  Hydrophobic  Hydrophobic | I470(2.60)  D474(2.64)  G493(3.24)  C494(3.25)  T496(3.27)  A497(3.28) | 3.5 Å  3.5 Å  3.0 Å  3.6 Å  3.8 Å  3.7 Å |
|  |  | Active | Induced dipole  Hydrophobic  Hydrophobic  Hydrophobic  Hydrophobic  Hydrophobic  Hydrophobic | D474(2.64)  G493(3.24)  C494(3.25)  A497(3.28)  K565(ECL2)  I568(ECL2)  C569(ECL2) | 3.7 Å  3.2 Å  3.2 Å  3.0 Å  3.5 Å  3.3 Å  2.9 Å |
| V502A | 3.33 | Inactive | Hydrophobic | Y563(ECL2) | 3.5 Å |
|  |  | Active | Polar  Hydrophobic  Hydrophobic | C549(4.53)  Y563(ECL2)  P571(ECL2) | 3.9 Å  3.6 Å  3.9 Å |
| M527T | ICL2 | Inactive | - | - | - |
|  |  | Active | - | - | - |
| Y582AF | 5.39 | Inactive | HB (side chain-backbone)  Hydrophobic  Hydrophobic  Hydrophobic  Induce dipole/hydrophobic  HB (backbone-backbone) | L552(4.56)  L555(4.59)  P556(4.60)  I560(ECL2)  S561(ECL2)  L578(ECL2) | 2.7 Å  3.9 Å  3.8 Å  3.9 Å  3.3 Å  3.4 Å |
|  |  | Active | HB (side chain-backbone)  Hydrophobic  Induce dipole  Induce dipole/hydrophobic  Polar | L552(4.56)  L555(4.59)  P556(4.60)  S561(ECL2)  L578(ECL2) | 2.8 Å  3.1 Å  3.8 Å  3.5 Å  3.7 Å |
| A593P/V | 5.50 | Inactive | Hydrophobic | V509(3.40) | 3.7 Å |
|  |  | Active | Induce dipole | T513(3.44) | 3.4 Å |
| F594I | 5.51 | Inactive | Aromatic | F634(6.45) | 2.9 Å |
|  |  | Active | Aromatic | F634(6.45) | 3.6 Å |
| R625A | 6.36 | Inactive | Repulsive/Polar  Hydrophobic  Hydrophobic | R450(2.40)  L677(7.52)  F681(7.56) | 3.6 Å  3.3 Å  3.8 Å |
|  |  | Active | - | - | - |
| F634I | 6.45 | Inactive | Aromatic | F594(5.51) | 2.9 Å |
|  |  | Active | Aromatic | F594(5.51) | 3.6 Å |
| A638V | 6.49 | Inactive | - | - | - |
|  |  | Active | - | - | - |
| F642I | 6.53 | Inactive | - | - | - |
|  |  | Active | - | - | - |
| Y643A | 6.54 | Inactive | HB (backbone-side chain)  Hydrophobic | K660(7.35)  L663(7.38) | 3.3 Å  3.0 Å |
|  |  | Active | Hydrophobic | L663(7.38) | 2.9 Å |
| A644V | 6.55 | Inactive | HB (backbone-side chain) | K660(7.35) | 3.3 Å |
|  |  | Active | - | - | - |
| I648V | 6.59 | Inactive | Induce dipole/hydrophobic  Hydrophobic  HB (backbone-backbone) | D573(ECL2)  I583(5.40)  N650(ECL3) | 3.6 Å  2.9 Å  3.5 Å |
|  |  | Active | Hydrophobic  HB (backbone-backbone)  Induce dipole | I583(5.40)  N650(ECL3)  K651(ECL3) | 3.2 Å  3.4 Å  3.3 Å |
| L665V | 7.40 | Inactive | - | - | - |
|  |  | Active | - | - | - |

BW = Ballesteros-Weinstein numbering

HB = hydrogen bonds (s.c. = side chain; b.b. = backbone)

References in Table 3C
